# Unprecedented loss of ammonia assimilation capability in a urease-encoding bacterial mutualist

**DOI:** 10.1186/1471-2164-11-687

**Published:** 2010-12-02

**Authors:** Laura E Williams, Jennifer J Wernegreen

**Affiliations:** 1 The Institute for Genome Sciences and Policy, Duke University, Durham, NC, USA; 2 Nicholas School of the Environment, Duke University, Durham, NC, USA

## Abstract

**Background:**

*Blochmannia *are obligately intracellular bacterial mutualists of ants of the tribe Camponotini. *Blochmannia *perform key nutritional functions for the host, including synthesis of several essential amino acids. We used Illumina technology to sequence the genome of *Blochmannia *associated with *Camponotus vafer*.

**Results:**

Although *Blochmannia vafer *retains many nutritional functions, it is missing glutamine synthetase (*glnA*), a component of the nitrogen recycling pathway encoded by the previously sequenced *B. floridanus *and *B. pennsylvanicus*. With the exception of *Ureaplasma*, *B. vafer *is the only sequenced bacterium to date that encodes urease but lacks the ability to assimilate ammonia into glutamine or glutamate. Loss of *glnA *occurred in a deletion hotspot near the putative replication origin. Overall, compared to the likely gene set of their common ancestor, 31 genes are missing or eroded in *B. vafer*, compared to 28 in *B. floridanus *and four in *B*. *pennsylvanicus*. Three genes (*queA, visC *and *yggS*) show convergent loss or erosion, suggesting relaxed selection for their functions. Eight *B. vafer *genes contain frameshifts in homopolymeric tracts that may be corrected by transcriptional slippage. Two of these encode DNA replication proteins: *dnaX*, which we infer is also frameshifted in *B. floridanus*, and *dnaG*.

**Conclusions:**

Comparing the *B. vafer *genome with *B. pennsylvanicus *and *B. floridanus *refines the core genes shared within the mutualist group, thereby clarifying functions required across ant host species. This third genome also allows us to track gene loss and erosion in a phylogenetic context to more fully understand processes of genome reduction.

## Background

*Candidatus *Blochmannia species (hereafter, *Blochmannia*) are obligately intracellular, primary mutualists of ants belonging to the tribe Camponotini [1,2]. *Blochmannia *have been found in every species of *Camponotus *surveyed to date, as well as other genera within Camponotini, suggesting an ancient and stable association with the ant tribe [3]. The nutritional basis of the symbiosis was first suggested by the location of *Blochmannia *in specialized host cells intercalated among epithelial cells in the insect midgut [4,5]. Genome sequencing of *B*. *floridanus *and *B. pennsylvanicus *revealed biosynthetic pathways for all essential amino acids except arginine, further supporting the hypothesis that *Blochmannia *provides important nutritional functions to the ant host [6,7]. The symbiont also recycles nitrogen from urea into glutamine via the activity of urease and glutamine synthetase [8], which are encoded by the two previously published *Blochmannia *genomes [6,7]. Urease hydrolyzes urea to ammonia and carbon dioxide, and glutamine synthetase assimilates nitrogen from ammonia into glutamine, which feeds into other amino acid biosynthetic pathways [9]. This process may consume the nitrogenous waste products of the ant host and also urea in animal or bird waste collected and ingested by some *Camponotus *species [8].

Similar to other primary obligate mutualists of insects, *Blochmannia *genomes are extremely reduced in size (705-792 kb), show strong AT bias (27-29.5% GC content), and undergo accelerated rates of molecular evolution [6,7,10]. Comparative analyses of *Buchnera*, the primary mutualist of aphids, suggest that ongoing genome reduction proceeds primarily as a gradual erosion of individual genes, rather than by punctuated losses of large DNA segments encoding multiple genes [11]. Comparisons of the *B. floridanus *and *B. pennsylvanicus *genomes indicate a similar phenomenon in *Blochmannia *[7]. The accumulation of substitutions, small indels and frameshift errors slowly degrades genes, and the resulting pseudogenes are gradually eliminated from the genome by the underlying deletional bias in bacteria [12]. The complement of genes lost by an endosymbiont genome is determined in part by chance events, such as the fixation of deleterious mutations due to small effective population sizes, and by the historical contingencies of gene loss in ancestral genomes, which influence the strength of selective pressure on remaining genes [13]. Independent loss of the same gene from multiple lineages would suggest relaxed selection on gene function, rather than stochastic effects of drift.

The AT mutational bias observed in obligate intracellular symbionts results in a high density of homopolymeric tracts of adenines or thymines in the genome, with some tracts as long as 12 bp. These polyA or polyT tracts are prone to polymerase slippage during replication, which can introduce frameshift errors in the gene. In some cases, such frameshifts may constitute the initial events of gene erosion, leading to accumulation of mutations and eventual degradation to a pseudogene [14]. However, some frameshifts are conserved among geographically disparate symbiont populations and even between different species, with no detectable degradation of the gene [15,16]. Full-length, functional protein products are generated from these frameshifted genes as a result of transcriptional slippage in the homopolymeric tract, which produces a mixed mRNA pool of full-length and frameshifted transcripts [15].

To better understand the nutritional symbiosis between *Blochmannia *and Camponotini and processes of genome reduction in this endosymbiont, it is essential to compare multiple genomes. Expanding the set of completed *Blochmannia *genomes may shed light on the 'core' genes shared across *Blochmannia *and refine our knowledge of the functions that are required in this symbiosis. Comparing multiple genomes within this group can also clarify the *Blochmannia *pan-genome and let us reconstruct the likely gene content of ancestral genomes, thereby elucidating patterns of gene loss and erosion. Finally, analysis of *Blochmannia *genomes from different *Camponotus *host species may help us understand how this symbiosis evolves in diverse hosts that occupy different habitats and geographic ranges.

Here, we use Illumina technology to sequence the genome of *Blochmannia vafer*, the endosymbiont of *Camponotus vafer*. The addition of this genome to the two previously sequenced *Blochmannia *genomes lets us investigate genomic changes in a phylogenetic context. Also, *C. vafer *occupies a different habitat and geographic range compared to *C. floridanus *and *C. pennsylvanicus*. Whereas *C. pennsylvanicus *is widespread throughout much of the eastern and central United States, and *C. floridanus *is distributed throughout the southeastern United States as well as Central and South America [17], *C. vafer *is found in a more restricted range in the southwestern United States and northeastern Mexico [18]. Completion of the *B. vafer *genome allows us to investigate the nature of the mutualism in ecologically distinct *Camponotus *species.

## Results

### Shared *Blochmannia *genes include urease gene cluster

**Figure 1:**
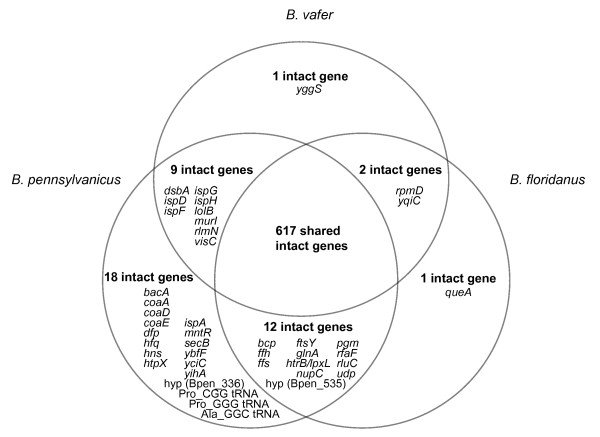
**Comparison of intact genes encoded by three *Blochmannia *genomes**. To highlight potential metabolic differences among genomes, only intact genes are shown. These include protein-coding and RNA-coding genes. Eroded pseudogenes in a given genome are considered missing due to loss of function. Genes with a single frameshift in a polyA or polyT tract are considered intact for this purpose, because transcriptional slippage may restore expression of a full-length protein product. In this comparison, *yidC *and *yidD *in *B. floridanus *are counted as a single gene, due to fusion in *B. pennsylvanicus *and *B. vafer*.

The *B. vafer *genome shares many features with the *B. pennsylvanicus *and *B. floridanus *genomes, including reduced size and low %GC content (Table 1). Genes shared among the three *Blochmannia *include 617 intact genes, 575 of which are protein-coding (Figure 1). For the purposes of these comparisons of functional capabilities, we include as 'intact' those genes that contain a single indel within a homopolymeric tract, since the reading frame may be restored via transcriptional slippage [15]. *B. vafer*, *B. floridanus *and *B. pennsylvanicus *encode the same set of DNA replication proteins and ribosomal proteins, with the exception of the 50 S ribosomal protein RpmD, which is absent in *B. pennsylvanicus*. All three *Blochmannia *encode the same pathways for essential amino acid biosynthesis and sulfate assimilation, the latter of which contributes to cysteine biosynthesis. A urease gene cluster, which includes the structural genes *ureABC *and the accessory genes *ureDFG *[19], is also present in all three genomes. Gene order and strand orientation is strictly conserved along all three *Blochmannia *genomes. The set of genes encoded by each *Blochmannia *genome is listed in Additional File 1.

**Table 1:** *Blochmannia *genome statistics.

	*B. vafer*	*B. floridanus*	*B. pennsylvanicus*
Size (bp)	722,593	705,557	791,654
GC content	27.5%	27.4%	29.6%
Total genes	631	637	659
Protein-coding genes	587	590^1^	610
tRNA	37	37	40^2^
rRNA	3	3	3
Other RNA	2	3^3^	3^3^
Pseudogenes	2	4	3
Average CDS length	1,006	1,002	995
Median IGS length	134	114	172
Percent protein-coding	81.7%	83.9%	76.7%
Percent coding (inc. RNA)	82.9%	85.0%	77.8%

^1 ^Gene counts for *B. floridanus *include four protein-coding genes not identified in the original annotation that were detected by comparisons with *B. vafer *and *B. pennsylvanicus*.

^2 ^Re-analysis of the *B. pennsylvanicus *genome revealed that the Gly-GGA tRNA pseudogene reported earlier is likely functional.

^3 ^We identified a RNA-coding gene in *B. floridanus *and *B. pennsylvanicus *that was not previously annotated.

### Loss of glutamine synthetase in *B. vafer*

The most recent common ancestor of all three *Blochmannia *had at least 660 genes (Figure 1). This reconstruction considers only genes that are intact in at least one of the genomes (the *uvrD *pseudogene found in *B. pennsylvanicus *is therefore not included). At least 31 genes were lost or eroded along the branches leading from the common ancestor to *B. vafer*, compared to 28 genes for *B. floridanus *and four for *B. pennsylvanicus*. Twelve genes are missing or eroded only in *B. vafer *(Figure 1). Of these, glutamine synthetase (*glnA*) is perhaps the most startling. We confirmed the absence of *glnA *in *Blochmannia *from eight additional *C. vafer *colonies by Sanger sequencing of this region. The sequences were identical, with the exception of one substitution and three indels. Analysis of the *trpE *gene encoded by *B. vafer *revealed three distinct genotypes, demonstrating that *glnA *is missing from different *B. vafer *strains.

In other *Blochmannia *species, Class I glutamine synthetase is thought to act in concert with the urease gene cluster (*ureDABCFG*) to recycle nitrogen into amino acid biosynthesis [8]. Urease converts urea to carbon dioxide and ammonia, the latter of which is then assimilated into glutamine by glutamine synthetase. Despite losing *glnA, B. vafer *retains *ureDABCFG*, which have high amino acid sequence identity (53-80%) to the *B. floridanus *urease proteins. Loss of glutamine synthetase in *B. vafer *disrupts the ammonia assimilation step of nitrogen recycling. In *E. coli*, glutamate dehydrogenase (*gdhA*) encodes an alternative ammonia assimilation pathway [20], but none of the sequenced *Blochmannia *retain this gene. We searched the NCBI Genomes database to determine whether any other completely sequenced bacterium possesses urease but lacks glutamine synthetase and glutamate dehydrogenase. Of the 371 bacterial genomes encoding urease, 359 also have an ortholog of the *B. pennsylvanicus *Class I glutamine synthetase. Among the remaining 12 genomes, four have an ortholog of *glnA *from *Bacteroides fragilis*, which is the prototypical example of a Class III glutamine synthetase, and five genomes have an ortholog of *E. coli *glutamate dehydrogenase (*gdhA*). In all, 368 of the 371 bacterial genomes with urease also encode an ammonia assimilation pathway catalyzed by either glutamine synthetase (Class I or Class III) or glutamate dehydrogenase. The three genomes lacking *glnA *or *gdhA *are all strains of *Ureaplasma*, which uses high intracellular concentrations of ammonia to generate an electrochemical gradient for driving ATP synthesis [21].

### The urease gene cluster in *Blochmannia*

To determine whether loss of glutamine synthetase is correlated with relaxed selection on the urease genes in *B. vafer*, we compared evolutionary rates along the lineages leading to *B*. *vafer *and *B. floridanus *since their divergence from a common ancestor. For the 570 orthologs analyzed, the fold-increase in evolutionary rate along the lineage leading to *B. vafer *had a wide range (0.1 - 4.6), but a median value of 1.04, indicating no systematic rate difference between the *B. vafer *and *B. floridanus *lineages (Additional File 2). At urease genes, the rate increase in *B. vafer *varied from 0.53 - 1.87 for the three structural genes (*ureABC*) and 0.96 - 1.19 for the accessory genes (*ureDFG*), indicating that the absence of *glnA *in *B. vafer *is not correlated with consistent rate acceleration in the urease proteins.

We found no evidence to suggest that the urease operon in *Blochmannia *was acquired by horizontal gene transfer from outside of the gamma-proteobacteria. *Blochmannia *urease proteins were most similar by BLASTP analysis to those of other gamma-proteobacteria, including *Pseudomonas *(e.g., NC_010501) and *Klebsiella *(e.g., NC_009648). Gene order in the urease gene cluster was conserved among the related free-living bacteria and *Blochmannia*; however, the *Pseudomonas *urease operon consists of eight genes (*ureDABCEJFG*), whereas the *Blochmannia *urease operon lacks urease accessory genes *ureE *and *ureJ*. For all three sequenced *Blochmannia*, the intergenic sequence (IGS) between *ureC *and *ureF *is 298 - 470 bp, much larger than the other IGSs in this operon, which ranged from 2 - 122 bp. As described below, the longer IGSs suggest that *ureE *and *ureJ *might have been present in the ancestor of *Blochmannia*.

### Processes of genome reduction in *B. vafer*

#### (i) Deletion hotspot near the origin of replication

**Figure 2:**
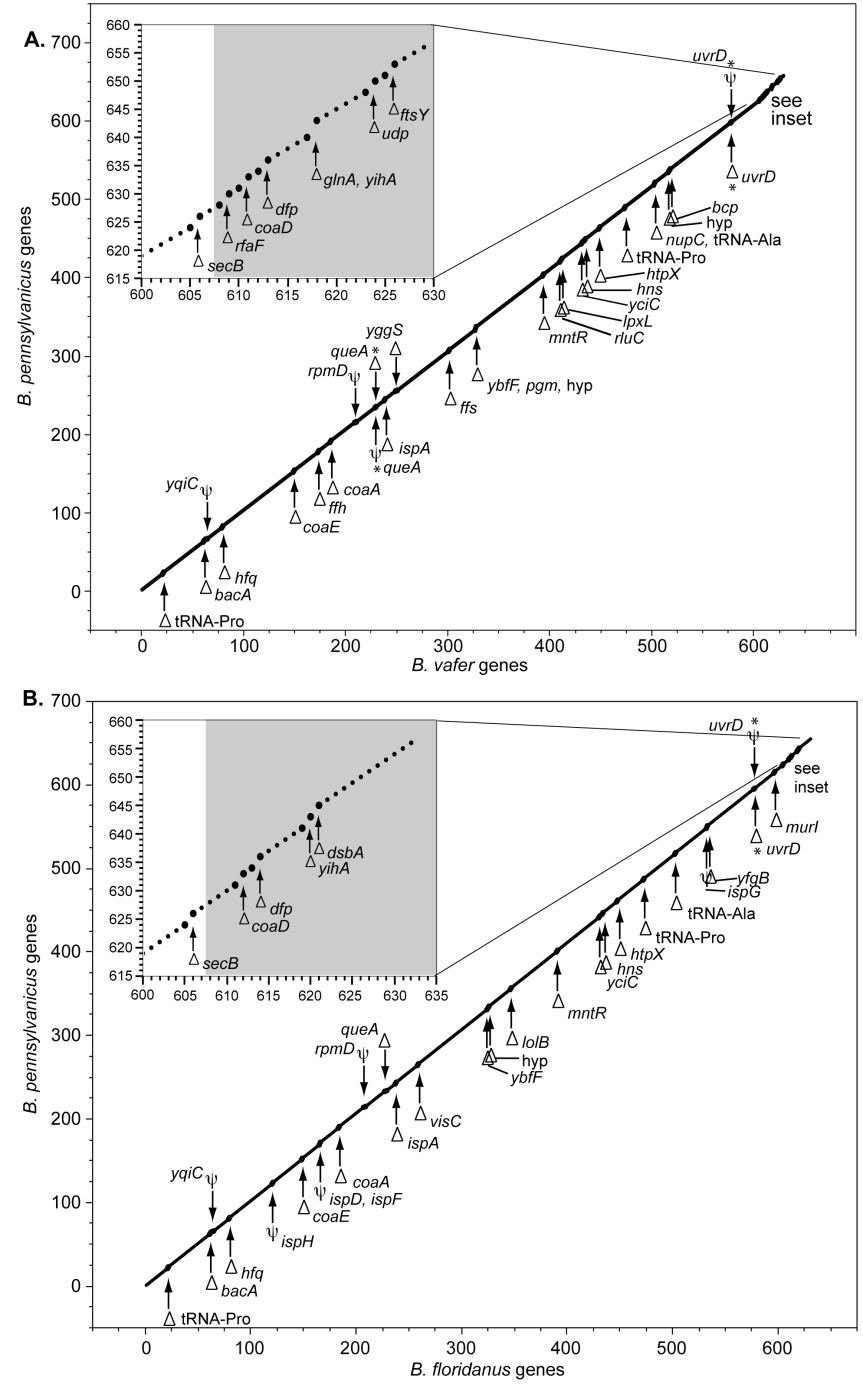
**Gene loss in *B. vafer *and *B. floridanus *compared to *B. pennsylvanicus***. (a) *B. vafer *genes were plotted against orthologs in *B. pennsylvanicus*. The x- and y-axes correspond to gene number in *B. vafer *and *B. pennsylvanicus*, respectively. Locations of missing genes (represented by a delta symbol) and eroded genes (represented by a psi symbol) are emphasized by larger dots. Gene names are listed next to the symbols. Two genes, marked with asterisks, are missing in one of the genomes and present only as a pseudogene in the other genome. The inset shows a more detailed view of the ~30 kb region in *B. vafer *near the putative replication origin, in which eight genes are missing compared to *B. pennsylvanicus*. The grey shaded region in the inset indicates the relocation of the origin in *B. vafer *compared to *B. pennsylvanicus*. (b) *B*. *floridanus *genes were plotted against orthologs in *B. pennsylvanicus*, using the same methodology as above.

**Figure 3:**
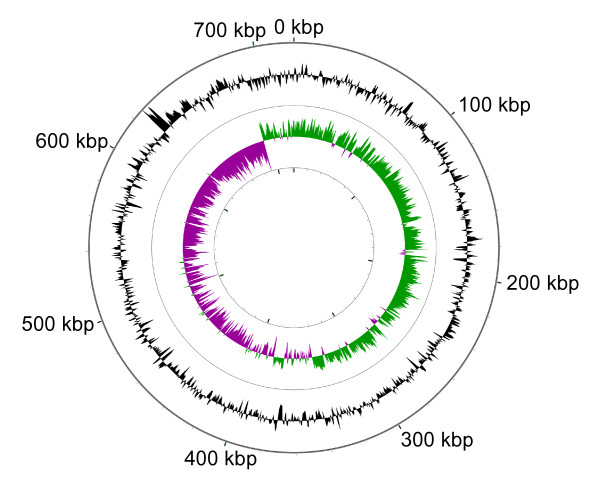
**GC content and GC skew of *B. vafer *genome**. GC content (black) and GC skew (green and purple) of the *B. vafer *genome. For GC skew, the center line indicates the average GC skew value for the genome. Green shading above the line denotes GC skew values greater than the genome average, whereas purple shading below the line denotes GC skew values less than the genome average. Major and minor tick marks on the outermost and innermost circles show nucleotide position on the genome in 100 kbp and 20 kbp increments, respectively.

Genes missing or eroded in *B. vafer *were generally scattered throughout the genome, with the exception of eight missing genes near the putative origin of replication (Figure 2). Because of the absence of a characteristic cluster of *dnaA *boxes, we identified the origin of replication based on the shift in GC skew occurring between *yibN *and *hldD *(Figure 3). Similar to *B. floridanus*, the putative origin of *B. vafer *is located approximately 32 kb upstream of *gidA*, which is the origin of replication of *B. pennsylvanicus *[6,7]. This 32 kb region contains three genes that are missing from both *B. vafer *and *B. floridanus *and were likely lost prior to their divergence, another gene missing from *B. floridanus *only, and four genes missing from *B. vafer *only, including *glnA*.

#### (ii) Frameshifts within homopolymeric tracts

**Figure 4:**
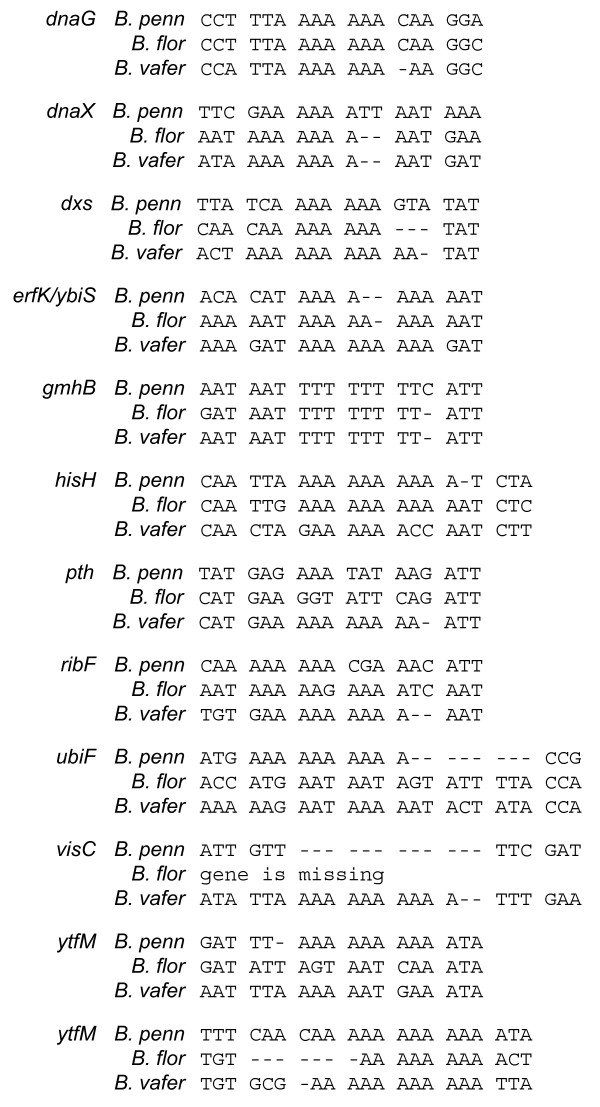
**Frameshifts in polyA or polyT tracts in *Blochmannia *genes**. Alignments illustrate frameshifted regions of the eleven genes that contain indels within homopolymeric tracts in one or more *Blochmannia *genomes. Apart from the frameshifts shown, these genes are otherwise in frame. The frameshifts may be corrected by transcriptional slippage to yield full-length, in-frame transcripts.

Similar to other obligate insect endosymbionts, the genome of *B. vafer *is extremely AT-biased (Table 1). All three *Blochmannia *genomes contain numerous long (9 or more bp) polyA tracts within protein-coding regions (153 such tracts in *B. vafer*, compared to 134 in *B*. *floridanus *and 114 in *B. pennsylvanicus*). Indels in homopolymeric tracts and resulting frameshifts are considered key events in gene inactivation and loss in endosymbiont genomes [14]. However, some frameshifts may be corrected by polymerase slippage during transcription, allowing expression of full-length proteins [7,15,16]. Eight *B. vafer *genes have short indels within polyA or polyT tracts resulting in frameshifts, compared to four such genes in *B*. *floridanus *and four in *B. pennsylvanicus *(Figure 4). For the four genes that were frameshifted exclusively in *B. vafer *(*dnaG, dxs, pth *and *ribF*), we found no evidence of unusually accelerated evolutionary rates along the lineage leading to *B. vafer*. Specifically, when compared to the lineage leading to *B. floridanus*, the fold-increase in evolutionary rates at the four genes in *B*. *vafer *ranged from 1-1.5, well within the distribution for the 570 orthologs tested. Although it is conceivable that these frameshifts are recent, it is also possible that the reading frame is corrected via transcriptional slippage, as demonstrated for other *Blochmannia *and *Buchnera *genes [7,15,16].

Of the eight *B. vafer *genes with frameshifts, three are involved in information transfer: *dnaX, dnaG *and *pth*. *DnaX *encodes two subunits of the DNA polymerase III holoenzyme, which is the primary replicative enzyme in bacteria. In *E. coli*, expression of full-length *dnaX *generates the tau subunit, whereas ribosomal slippage generates the shorter gamma subunit [22-24]. Both of these subunits are thought to participate in assembly of the beta sliding clamp, which affects processivity of the pol III holoenzyme [25]. The *dnaX *frameshift reported here occurs downstream of the ribosomal slippage site, within the region exclusive to the tau subunit. Comparisons of *B. vafer *and *B. floridanus *allowed us to identify the same frameshift in *B*. *floridanus dnaX*, which was previously reported as truncated [6]. Because this frameshift may be corrected by transcriptional slippage, we propose that expression of full-length *dnaX *tau subunit occurs at some level in both *B. vafer *and *B. floridanus*. This frameshift is not found in *B*. *pennsylvanicus dnaX*, possibly because the polyA tract is interrupted by two thymines, which reduces the chances of slippage during replication or transcription (Figure 4). The two other frameshifted information transfer genes in *B. vafer *encode DNA primase (*dnaG*) and peptidyl-tRNA hydrolase (*pth*) (Figure 4). DnaG synthesizes short RNA primers essential for lagging strand DNA replication, whereas Pth plays an important role in maintaining efficient protein synthesis by cleaving peptidyl-tRNAs released from stalled ribosomes [26].

**Figure 5:**
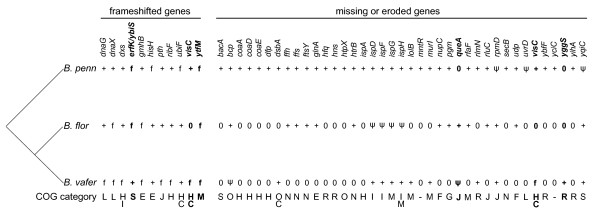
**Gene loss and erosion in *Blochmannia***. The differential gene content of the three *Blochmannia *genomes is shown in a phylogenetic context to highlight small frameshifts and missing or eroded genes. Presence of a gene is shown with a plus sign and absence of a gene with a zero. Pseudogenes are indicated by a psi symbol (ψ), and frameshifts are denoted with the letter f. COG functional categories are shown on the bottom line. Genes for which frameshifts, erosion, or loss occur independently in different *Blochmannia *lineages are marked in boldface.

To provide a phylogenetic perspective on these indels within homopolymeric tracts, we confirmed that *B. vafer *and *B. floridanus *are more closely related to each other than either is to *B. pennsylvanicus *(Additional File 3). Based on this topology, two genes, *erfK *and *ytfM*, show evidence for independent frameshift events and/or a reversion to an intact reading frame (Figure 5). Independent frameshift events in *ytfM *are supported by the different location of the indel in *B*. *pennsylvanicus*, compared to *B. floridanus *and *B. vafer *(Figure 4).

#### (iii) Convergent patterns of gene loss or erosion among *Blochmannia*

In addition to examining the history of frameshifted genes, we also identified several examples of convergent gene loss and erosion among the three *Blochmannia *genomes (Figure 5). Three genes (*queA*, *visC *and *yggS*) were likely lost or eroded in independent events. *QueA *was likely lost in the lineage leading to *B. pennsylvanicus *and degraded in the lineage leading to *B*. *vafer *sometime after *B. vafer *and *B. floridanus *diverged. *VisC *is intact in *B. pennsylvanicus *but absent from *B. floridanus*. In *B. vafer*, *visC *has a single indel within a tract of 11 adenines. This frameshift might be corrected by transcriptional slippage, but it could, in principle, also represent very recent gene erosion that is independent from *visC *loss in *B. floridanus*.

In the case of *yggS*, no detectable relicts of the gene remain in either *B. floridanus *or *B*. *pennsylvanicus*, but the gene is intact in *B. vafer*. We found no evidence to support the hypothesis that *yggS *was acquired by *B. vafer *via horizontal gene transfer. The top BLASTP hits for *B. vafer *YggS were other gamma-proteobacteria, including the insect endosymbiont *Sodalis glossinidius*, which is considered a close relative of *Blochmannia *[27]. In addition, the corresponding intergenic spacer sequences in *B. floridanus *(663 bp) and *B. pennsylvanicus *(942 bp) were much larger than median IGS lengths (114 bp and 172 bp, respectively; see below), suggesting the presence of a severely eroded gene remnant. Finally, the GC content and codon usage of *yggS *is typical for the *B. vafer *genome (data not shown). Combined, this data strongly argues against recent horizontal transfer and are most consistent with *yggS *being present in the ancestral *Blochmannia *genome. This gene was most likely lost independently in the lineage leading to *B. floridanus*, sometime after *B. floridanus *and *B. vafer *diverged, and in the lineage leading to *B. pennsylvanicus*.

**Figure 6:**
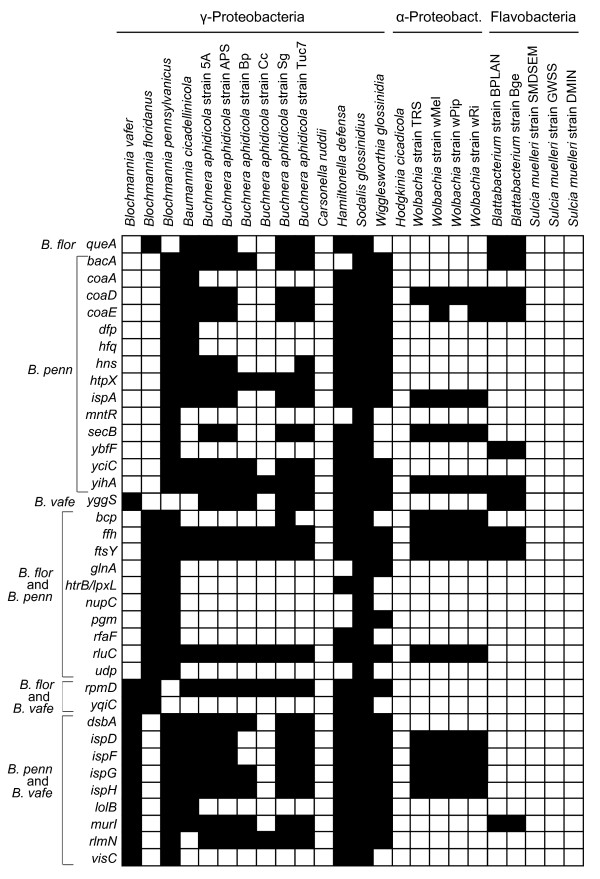
**Gene content differences among select bacterial endosymbionts of insects**. For each gene, the protein sequence from *E. coli *K12 MG1655 (NC_000913) was compared to endosymbiont genomes using TBLASTN. Black squares indicate presence of the gene, and white squares indicate that the gene is either missing or a pseudogene.

YggS might be involved in osmotolerance [28]; however, its exact function is unclear [29]. Notably, this protein is encoded by a number of other bacterial endosymbionts of insects (Figure 6), including *Blattabacterium *(cockroach), *Buchnera aphidicola *(aphid), *Hamiltonella defensa *(aphid), *Sodalis glossinidius *(tsetse fly), and *Wigglesworthia glossinidia *(tsetse fly). Of those endosymbionts lacking *yggS*, a few have severely reduced genomes, such as *Carsonella *(160 kb), *Hodgkinia *(144 kb) and *Sulcia *(244-277 kb).

Although the three *Blochmannia *genomes are severely reduced, most genome reduction occurred before their divergence. Ongoing gene disruption and loss since they diverged has affected ‹10% of genes encoded in the ancestral genome. Given this, the probability that continued gene erosion and loss would occur by chance in two different *Blochmannia *lineages at the same gene is rather low. Instead, independent loss or erosion, such as we observed at *queA *and *yggS*, suggests relaxed selective pressure on gene function. Conversely, the retention of *queA *and *yggS *in *B. floridanus *and *B. vafer*, respectively, suggests that their functions may remain important for host-specific aspects of the symbiosis.

#### (iv) Longer intergenic sequences mark locations of gene losses

Intergenic sequences in *B. vafer *and *B. floridanus *were significantly shorter than those of *B. pennsylvanicus *(Wilcoxon rank sum test; p ‹ 0.001) (Table 1). Because gene order is strictly conserved in all three sequenced *Blochmannia *genomes, we were able to identify IGSs spanning missing genes in each genome. For both *B. vafer *and *B. floridanus*, such IGSs were significantly longer than IGSs between conserved genes (Wilcoxon rank sum test; p ‹0.0001). We detected no significant difference in the lengths of IGSs spanning missing genes between *B. vafer *and *B*. *floridanus *(Wilcoxon rank sum test; p ›0.05), suggesting that deletion rates in these IGSs are similar in both genomes.

The importance of intergenic sequences in reduced endosymbiont genomes is largely unexplored. Coding sequences, including protein-coding and RNA-coding genes, occupy from 77.8 - 85.0% of the three *Blochmannia *genomes (Table 1), a lower fraction than many free-living bacteria [12]. Non-coding elements within IGSs may play important roles in fundamental cellular processes. We searched the *Blochmannia *genomes for long palindromic sequences, which may impact control of transcription or mRNA stabilization [30]. We identified 20 large (›30 bp) palindromes in *B. floridanus*, 10 in *B. vafer *and 6 in *B. pennsylvanicus*; all but two were located at least partially in IGSs. Palindrome lengths ranged from 64 - 136 bp in *B. vafer *(median 89 bp), including four exceptionally long palindromes ›100 bp in length. *B. floridanus *and *B. pennsylvanicus *palindromes ranged from 62 - 104 bp (median 76 bp) and 62 - 78 bp (median 72 bp), respectively. Given the high rates of nucleotide and indel mutations in *Blochmannia *[31], intergenic palindromes would likely decay in the absence of selection favoring their presence.

## Discussion

**Figure 7:**
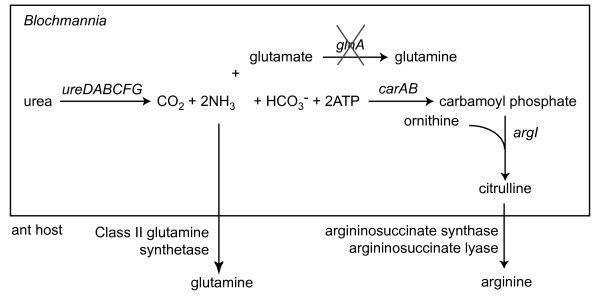
**Possible alternative pathways for ammonia assimilation in *B. vafer***. Three possible pathways for ammonia assimilation in *Blochmannia *are shown. Glutamine synthetase (*glnA*) is crossed out because of the absence of *glnA *in *B. vafer*.

Like *B. floridanus *and *B. pennsylvanicus*, *B. vafer *retains several key nutritional functions that may benefit the ant host, including biosynthesis of all essential amino acids except arginine. However, the nitrogen recycling pathway proposed for *B. floridanus *and *B*. *pennsylvanicus *is not complete in *B. vafer *(Figure 7). Although *B. vafer *encodes the urease gene cluster, which hydrolyzes urea to ammonia and carbon dioxide, it lacks glutamine synthetase or any other means of assimilating nitrogen from ammonia into glutamine, an important precursor to biosynthesis of other amino acids.

In *B. vafer*, ammonia might be exported into the host cell cytosol, where Class II glutamine synthetase encoded by the ant host [32] may catalyze the assimilation of nitrogen and prevent the toxic accumulation of ammonia (Figure 7). Alternatively, excess ammonia may be used as a nitrogen donor by carbamoyl phosphate synthase (*carAB*). This enzyme, along with *argI*, catalyzes the initial steps of arginine biosynthesis in all three sequenced *Blochmannia *(Figure 7). The resulting product, citrulline, is exported to the host cell cytosol to complete arginine biosynthesis [6]. Although glutamine is the preferred nitrogen donor for *carAB*, ammonia can be used as a substrate in the absence of glutamine [33]. Whether carbamoyl phosphate synthase plays a role in eliminating ammonia in *B. vafer *depends upon the concentration of glutamine in the endosymbiont cells (i.e., the amount of glutamine imported from the ant host). If glutamine is limiting, then *carAB *may use the excess ammonia produced by urease as a substrate.

With the exception of *Ureaplasma*, *B. vafer *is the only completely sequenced bacterium in the current NCBI Genomes database that possesses urease but lacks the ability to assimilate ammonia into glutamine or glutamate. All other bacteria with urease also encode ammonia assimilation pathways catalyzed by either glutamine synthetase (Class I or, more rarely, Class III) or glutamate dehydrogenase. This includes the cockroach endosymbiont *Blattabacterium*, which encodes urease and glutamate dehydrogenase [34,35]. The only exceptions are strains of the bacterial genus *Ureaplasma*, a commensal and opportunistic pathogen that colonizes the human urogenital tract and belongs to the Class Mollicutes [36]. In *Ureaplasma*, high intracellular concentrations of ammonia are thought to generate an electrochemical gradient that drives ATP synthesis [21]. There is no evidence that this process occurs in *Blochmannia*. With the loss of glutamine synthetase, *B. vafer *may have relinquished a component of the ancestral mutualism to the ant host. It is unclear whether or how the loss of *glnA *affects the fitness of *B*. *vafer *and/or its ant host.

Gene loss and erosion is accelerated in the lineages leading to *B. vafer *and *B. floridanus *compared to *B. pennsylvanicus*. The mechanism(s) underlying the more extensive genome reduction in *B. vafer *and *B. floridanus *may occur at both the level of the endosymbiont and the level of the ant host. For example, the smaller size of intergenic spacers in *B. vafer *and *B*. *floridanus *suggest a faster rate of deletion mutations compared to the lineage leading to *B. pennsylvanicus*. Elevated mutation may reflect differences in the natural history of the host species sampled. Whereas *C. pennsylvanicus *and relatives in the subgenus Camponotus typically live in temperate regions and hibernate for part of the year, *C. vafer *and *C. floridanus *and their relatives live in warmer climates and their colonies are more active throughout the year. These differences in host activity and life history could affect the number of symbiont replications per year and thus influence the mutation rate per unit time.

Many genes missing in one or more *Blochmannia *genomes are also missing from other insect endosymbionts (Figure 6), suggesting that the functions encoded are not essential across all intracellular mutualists. Five genes showed evidence of independent instances of gene loss, erosion and/or frameshifts within *Blochmannia*. Particularly in the case of gene absence or erosion, these convergent patterns suggest relaxed selective pressure on gene function, rather than chance loss. These independent events may reflect the historical contingency of gene loss and erosion in the ancestral genome, which affects selective pressure on remaining genes.

We found frameshift errors in three genes involved in DNA replication or protein synthesis: *dnaG, dnaX *and *pth*. These genes are considered essential for fundamental cellular processes in *E. coli*. Although expression of full-length proteins may be restored by transcriptional slippage, previous work showed that full-length transcripts accounted for ‹30% of the transcripts produced from frameshifted genes [15]. Therefore, it is possible that frameshifts lower the effective expression of these genes in *B. vafer*. The impact on DNA replication fidelity and efficiency of protein synthesis is unclear.

The deletion hotspot detected around the putative origin of replication in both *B. vafer *and *B. floridanus *occurs in the 32 kb region between the *B. vafer *origin and the location of the origin in *B. pennsylvanicus*. Given that the putative origin of replication in *B. pennsylvanicus *occurs at the same genomic location as the origins in *E. coli *and in other insect endosymbionts of the gamma-proteobacteria, it seems likely that the origin of replication shifted in the ancestral lineage of *B. vafer *and *B. floridanus*. Although the mechanisms underlying this shift are unclear, it is interesting that relocation of the origin is correlated with a concentration of gene deletions in this region.

## Conclusions

Comparisons of the *B. vafer *genome with the previously published *B. floridanus *and *B*. *pennsylvanicus *genomes clarified the core set of genes and functions shared by this mutualist group. More broadly, these data inform studies of processes of genome reduction in intracellular bacteria. (i) The surprising loss of glutamine synthetase (*glnA*) from *B. vafer*, despite retention of urease genes, suggests a profound difference in nitrogen recycling within this species. In *B*. *floridanus *and *B. pennsylvanicus*, the ammonia generated by urease is assimilated during glutamine biosynthesis. In the *C. vafer*-*Blochmannia *association, ammonia assimilation may occur by an alternative pathway, such as glutamine biosynthesis in the host or the initial steps of arginine biosynthesis catalyzed by the endosymbiont. Database searches showed that lack of ammonia assimilation by glutamine synthetase or glutamate dehydrogenase in a urease-encoding bacterium is extremely rare. (ii) Evaluating *Blochmannia *genome reduction in a phylogenetic context revealed instances of convergent gene loss or erosion at *queA, visC*, and *yggS*, which may be associated with changes in selective pressure on these genes. (iii) A deletion hotspot in both *B. vafer *and *B. floridanus *occurs within a 32 kb region corresponding to the likely relocation of the replication origin. (iv) Eight *B. vafer *genes contain frameshifts in homopolymeric tracts, including two genes encoding DNA replication proteins and one gene important for efficient protein synthesis. Although such frameshifts may be corrected by transcriptional slippage, expression of full-length proteins is likely reduced compared to wild-type levels and has possible implications for efficiency and accuracy of information transfer.

## Methods

### *Blochmannia vafer *genomic DNA preparation

*B. vafer *genomic DNA (gDNA) was prepared from *C. vafer *workers and larvae collected from a single colony (Cvafe543) in the Coronado National Forest near Portal, Arizona. Vouchers from this colony will be deposited at the Bohart Museum of Entomology, University of California, Davis. We isolated *Blochmannia *cells from 7.8 g of ants using a Percoll density gradient centrifugation protocol described previously [37], with a 100 μm filtration step instead of the 90 μm filtration. Isolated *Blochmannia *cells were treated with DNase to remove extracellular host DNA, lysozyme and proteinase K to break down bacterial cell walls and membranes, and RNase to remove rRNA and other RNA species. After these treatments, we extracted gDNA with phenol chloroform followed by ethanol precipitation.

### Genome sequencing and assembly

A paired-end sequencing library was constructed at Illumina (Hayward, CA) following standard procedures. Adaptors were used to multiplex this sample with two other samples on a single Illumina lane. Sequencing on an Illumina Genome Analyzer II (GAII) with Illumina pipeline 1.4 produced 14,907,136 100-bp paired end reads.

We performed all data analysis in house. Prior to *de novo *assembly, we filtered the read dataset to remove read pairs in which either read had an ambiguous base call (denoted by N) or an average quality score ‹Q25. The filter retained 10,157,316 reads (68% of total read dataset). From these filtered reads, we randomly selected 3 million read pairs. We then assembled the reads with Velvet version 0.7.55 [38] using the following parameters: hash length 49, expected coverage 200, coverage cutoff 25 and no scaffolding. This assembly strategy generated three contigs of *Blochmannia *genome sequence comprising 42,053 bp, 118,857 bp and 561,103 bp.

To close the three gaps, we first determined gap sizes by PCR amplification of the original gDNA using primers annealing to flanking regions in the assembled contigs. We closed the gap corresponding to the membrane-spanning repeat region of *tolA *with Sanger sequencing, using Phred/Phrap/Consed [39-41] to assemble and manually examine the sequence. Although the remaining two gaps were short, secondary structure arising from long palindromes impaired Sanger sequencing. Upon inspection, we found that palindromes had also impeded Velvet assembly, but the gaps could be manually closed with existing Illumina sequence data (Additional File 4).

To generate the finished genome, we used Mosaik [42] to align the full Illumina read dataset against the closed sequence. This step clarified the majority genotype (i.e., for each nucleotide position, the base called in the majority of reads), since *de novo *assembly used a subset of reads rather than the full read dataset. The Mosaik DupSnoop module identified 42% of the aligned read pairs as duplicates. Because most of these duplicates were likely generated during PCR amplification of the Illumina library, we removed duplicates from the final Mosaik alignment. We used Consed to view the alignment and recall the consensus sequence. Coverage of the finished genome (without duplicate read pairs) averaged 542×, calculated using the MosaikCoverage module. The finished genome is deposited in GenBank with the accession number CP002189.

### Gene prediction and annotation

The finished genome was submitted to the MANATEE annotation engine hosted by the Institute for Genome Sciences (IGS) at the University of Maryland School of Medicine for an initial automated annotation. MANATEE uses Glimmer for gene prediction, tRNAscan to identify tRNAs, and RNAmmer or Rfam to identify structural RNAs.

We manually curated the MANATEE annotation. An ORF was retained if it had at least one of the following pieces of evidence: a Blast-Extend-Repraze (BER) alignment scoring ‹10^-5 ^to a protein from another organism, a HMM hit or a Prosite hit. We used the gene name suggested by SwissProt for the homologous gene in *E. coli *to provide consistency with other proteobacterial genome annotations. For genes with no suggested name in SwissProt, such as hypothetical genes, the gene name given for the homologous gene in *B. floridanus *or *B*. *pennsylvanicus *was used.

We curated start sites on the basis of BER alignments to other, non-*Blochmannia *genomes. BER searches also identified genes that contain a frameshift within a polyA or polyT tract but otherwise resemble intact genes. Because these frameshifts may be corrected by transcriptional slippage, we consider these genes potentially functional and annotated them as intact ORFs. The GenBank annotation indicates the position of such frameshifts.

We used two approaches to identify uncalled genes and pseudogenes in *B. vafer*. First, we searched intergenic regions at least 30 bp in length using BLASTX and the NCBI Protein Reference Sequences database with default parameters except that the low complexity sequence filter was turned off. We considered hits covering at least 60% of the subject protein sequence and yielding an e-score of ‹10^-5 ^as evidence of an uncalled gene or a pseudogene. For two intergenic regions, the top BLASTX hits were *B. pennsylvanicus *and/or *B. floridanus*, but the alignments had multiple nonsense mutations and gaps. Based on the alignments and their location within the conserved gene order of *Blochmannia*, we identified these regions as pseudogenes of *bcp *and *queA*.

We also took advantage of synteny in *Blochmannia *to screen for additional pseudogenes in *B. vafer*. For any genes present in *B. floridanus *and/or *B. pennsylvanicus *but missing in *B*. *vafer*, we compared the corresponding *B. vafer *intergenic region to the gene in the other *Blochmannia *genome(s) using TBLASTX. Apart from *bcp *and *queA *as noted above, we found no sequence similarity pointing to other recognizable pseudogenes in *B. vafer*.

We used the tRNAscan-SE program to identify tRNAs in the genome [43]. This program flags a tRNA as a potential pseudogene if it has a primary structure (HMM) score ‹10 bits or a secondary structure score ‹5 bits. (The primary structure score reflects nucleotide sequence similarity to sequence profiles of tRNAs, whereas the secondary structure score reflects RNA folding into the characteristic cloverleaf structure.) Three tRNAs in *B. vafer *were flagged due to low primary structure scores. However, their secondary structure scores were 28, 33 and 43, well above the 5 bit cutoff, and the predicted secondary structures had the characteristic cloverleaf form. Because endosymbiont genomes have extremely low GC content, the primary structure score cutoff normally used for bacteria with more moderate GC content, such as *E. coli*, may not be appropriate for *Blochmannia*. Our examination of the three tRNAs flagged as possible pseudogenes suggest that these are functional tRNAs, and we have annotated them as such. Re-analysis of the *B. pennsylvanicus *genome, which has one reported pseudo-tRNA, revealed the same phenomenon of low primary structure score but high secondary structure score, therefore we consider this tRNA functional in this study.

### Screening for *glnA *in additional *B. vafer*

We confirmed the absence of glutamine synthetase (*glnA*) in *B. vafer *from *C. vafer *workers collected from eight additional colonies in the Coronado National Forest. From a total DNA prep of an individual worker, we amplified a region of the *B. vafer *genome using primers anchored in *gmk *and *polA*, which flank *rpoZ *and *glnA *in *B. floridanus *and *B. pennsylvanicus*. The resulting amplicons include the complete coding sequence of *rpoZ *and the intergenic sequence corresponding to the former location of *glnA*. We sequenced the amplified region using Sanger sequencing and assembled the reads with Phred/Phrap/Consed [39-41]. We also sequenced *trpE *from all of these *B. vafer *except strain 21. Accession numbers are: HQ593629-HQ593636 (*rpoZ *region) and HQ603678-HQ603684 (*trpE*).

### Identification of *B. vafer *replication origin

We used the Pattern Locator program [44] to search the *B. vafer *genome for *dnaA *boxes using the consensus sequence TTWTNCACA [45]. In the absence of a characteristic cluster of *dnaA *boxes, we used the CGview Server [46] to analyze the GC skew of the genome. The CGView Server calculates GC skew as (G-C)/(G+C) using sliding windows of 1000 bp and a step size of 10 bp. The CGView Server also calculated GC content using the same sliding window parameters.

### Palindrome analysis

We used the EMBOSS palindrome script to search the genomes of *B. vafer*, *B*. *floridanus*, and *B. pennsylvanicus *for palindromes between 30-1000 bp, with a maximum gap of 20 bp between inverted repeats and a maximum of 3 mismatches.

### Comparisons among *Blochmannia *genomes

#### (i) Gene content comparisons

We compared the gene content of the three *Blochmannia *genomes using a bidirectional best hit BLASTP analysis available through RAST (Rapid Annotation using Subsystem Technology) [47]. Pairwise comparisons of the genomes identified shared genes as bidirectional best hits. For genes yielding unidirectional best hits or fragmented hits, such as genes with frameshifts in homopolymeric tracts, we examined the annotations and performed additional BLASTP analyses to determine the presence or absence of the gene.

#### (ii) Detection of additional *B. floridanus *and *B. pennsylvanicus *genes

Based on comparisons of all three sequenced *Blochmannia*, we identified four protein-coding genes in *B*. *floridanus *and a RNA-coding gene in both *B. floridanus *and *B. pennsylvanicus *that were not previously annotated. We included these genes in our analyses here. To determine the start and stop coordinates of the protein-coding genes, we used TBLASTN to align the *B. vafer *or *B*. *pennsylvanicus *amino acid sequence to the *B. floridanus *nucleotide sequence. If the alignment did not include start and stop codons, we extended the coordinates to include the closest upstream ATG start codon and the downstream stop codon. To determine the coordinates of the RNA-coding gene, we used the Rfam sequence search.

#### (iii) Analysis of frameshifted genes

Genes containing single frameshifts within polyA or polyT tracts were compared to orthologs in the other *Blochmannia *genomes. Nucleotide sequences were translated, aligned with ClustalX and back-translated to obtain nucleotide sequence alignments. For the purposes of comparing evolutionary rates at these loci, the frameshifts within polyA tracts were corrected, and the genes were treated as intact.

#### (iv) Evolutionary rate comparison

We compared rates of protein evolution in the lineages leading to *B. vafer *and *B. floridanus*, using *B. pennsylvanicus *as an outgroup. First, we used the Reciprocal Smallest Distance (RSD) algorithm [48] to identify orthologs shared among the three genomes. By using stringent detection parameters in RSD, we included only those orthologs with robust alignments for dN calculations. (Reflecting the stringent criteria, RSD detected slightly fewer shared orthologs (570) than the 575 protein-coding genes detected in our direct comparisons of gene contents.) For each shared ortholog, we estimated the three pairwise dN values using a likelihood-based approach in PAML [49]. We then calculated the fold-increase in evolutionary rate for each gene along the lineage leading to *B. vafer *as K_1_/K_2_, in which K_1 _is the amount of nonsynonymous change along the branch leading to *B. vafer *from the most recent common ancestor with *B. floridanus*, and K_2 _is the amount of nonsynonymous change along the branch leading to *B. floridanus*.

#### (v) IGS analysis

We defined intergenic spacer sequences (IGS) as the sequences between protein-coding genes, RNA-coding genes or pseudogenes. Our calculation of IGS lengths does not include instances of overlapping gene coordinates that result in no intergenic sequence between the two genes. We used a perl script written by Sheri Simmons to extract intergenic sequences from the *B. vafer *genome.

### Comparison of gene content across insect endosymbionts

Among select other bacterial endosymbionts, we checked for the presence of protein-coding genes that were missing or eroded in the *Blochmannia *genomes. For each gene, the protein sequence from *E. coli *K12 MG1655 (NC_000913) was used as the query in a TBLASTN search against the endosymbiont genomes. For a gene to qualify as present, the alignment must cover at least 60% of the *E. coli *protein sequence and have an e-score ‹10^-5 ^(with the exception of *ftsY*, which required at least 50% coverage and an e-score ‹10^-5^). We examined the annotations of the endosymbiont genomes to ensure that the hits corresponded to homologous genes, rather than a similar gene from the same family (such as *ispA *and *ispB*). If the region corresponding to the alignment was not annotated in the endosymbiont genome, we did a BLASTP analysis of the encoded amino acid sequence against the SwissProt database and used the top hit to identify the gene.

### Analysis of urease and ammonia assimilation enzyme distribution in bacterial genomes

We used the *B. pennsylvanicus ureC *amino acid sequence in a TBLASTN search of the NCBI Genomes database with TaxID 2 to limit the search to bacterial genomes. This search returned 371 hits with at least 50% coverage and an e-score ‹10^-30^. We then did a TBLASTN search of these 371 bacterial genomes using the *B. pennsylvanicus glnA *amino acid sequence. The gene was considered present if the alignment had at least 50% coverage and an e-score ‹10^-30^. For three genomes, we discovered that *ureC *and *glnA *were encoded on different chromosomes, and for one genome, *ureC *was encoded on a plasmid whereas *glnA *was encoded on the chromosome. Genomes that did not encode an ortholog of *B. pennsylvanicus glnA *were searched for Class III glutamine synthetase from *Bacteroides fragilis *YCH46 (NC_006347) and glutamate dehydrogenase from *E. coli *K12 MG1655 (NC_000913) using TBLASTN.

### Statistical analysis

All statistical analyses used JMP version 8.

## Authors' contributions

LEW assembled and annotated the genome and performed comparative analyses. JJW conceived the study and performed the evolutionary rate comparison. LEW and JJW designed the comparative analyses and wrote the manuscript. Both authors read and approved the final manuscript.

## Supplementary Material

Additional File 1 **Ortholog table of the three *Blochmannia *genomes**. Table listing the annotated gene sets for all three available *Blochmannia *genomes. Click here for file

Additional File 2 **Evolutionary rates analysis**. Table listing the complete results of the evolutionary rates comparison. Click here for file

Additional File 3 **Phylogenetic analysis of *Blochmannia vafer***. Description of phylogenetic analyses of the three sequenced *Blochmannia*. Click here for file

Additional File 4 **Gap closure**. Detailed methods used to close two gaps in the *B. vafer *genome assembly. Click here for file
