# Supplementary material for: Unprecedented loss of ammonia assimilation capability in a urease-encoding bacterial mutualist
Source: BMC Genomics. 2010 Dec 2;11:687. doi: 10.1186/1471-2164-11-687 (PMC3017870; doi:10.1186/1471-2164-11-687)
Supplement: Additional File 1 — Ortholog table of the three Blochmannia genomes. Table listing the annotated gene sets for all three available Blochmannia genomes. [file 1471-2164-11-687-S1.DOC]

**Additional file 1.**

**Ortholog table of the three *Blochmannia*** genomes.

| *B. vafer* | locus tag | *B. floridanus* | locus_tag | *B. pennsylvanicus* | locus_tag |
| --- | --- | --- | --- | --- | --- |
| *mnmG* | BVAF_001 | *gidA* | Bfl001 | *gidA* | BPEN_001 |
| *atpB* | BVAF_002 | *atpB* | Bfl002 | *atpB* | BPEN_002 |
| *atpE* | BVAF_003 | *atpE* | Bfl003 | *atpE* | BPEN_003 |
| *atpF* | BVAF_004 | *atpF* | Bfl004 | *atpF* | BPEN_004 |
| *atpH* | BVAF_005 | *atpH* | Bfl005 | *atpH* | BPEN_005 |
| *atpA* | BVAF_006 | *atpA* | Bfl006 | *atpA* | BPEN_006 |
| *atpG* | BVAF_007 | *atpG* | Bfl007 | *atpG* | BPEN_007 |
| *atpD* | BVAF_008 | *atpD* | Bfl008 | *atpD* | BPEN_008 |
| *atpC* | BVAF_009 | *atpC* | Bfl009 | *atpC* | BPEN_009 |
| *glmU* | BVAF_010 | *glmU* | Bfl010 | *glmU* | BPEN_010 |
| *mnmE* | BVAF_011 | *trmE* | Bfl011 | *trmE* | BPEN_011 |
| *yidCD* | BVAF_012 | *yidC* | Bfl012 | *yidCD* | BPEN_012 |
|  |  | *yidD*1 | Bfl013 |  |  |
| *rnpA* | BVAF_013 | *rnpA* | Bfl014 | *rnpA* | BPEN_013 |
| *rpmH* | BVAF_014 | *rpmH* | Bfl015 | *rmpH* | BPEN_014 |
| *dnaN* | BVAF_015 | *dnaN* | Bfl016 | *dnaN* | BPEN_015 |
| *gyrB* | BVAF_016 | *gyrB* | Bfl017 | *gyrB* | BPEN_016 |
| *ibpA* | BVAF_017 | *ibpA* | Bfl018 | *ibpA* | BPEN_017 |
| BVAF_018 | BVAF_018 | unknown | Bfl019 | BPEN_018 | BPEN_018 |
| *glyQ* | BVAF_019 | *glyQ* | Bfl020 | *glyQ* | BPEN_019 |
| *glyS* | BVAF_020 | *glyS* | Bfl021 | *glyS* | BPEN_020 |
| *sodA* | BVAF_021 | *sodA* | Bfl022 | *sodA* | BPEN_021 |
|  |  |  |  | tRNA-Pro | BPEN_022 |
| *prlC* | BVAF_022 | *prlC* | Bfl023 | *prlC* | BPEN_023 |
| *pit* | BVAF_023 | *pitA* | Bfl024 | *pitA* | BPEN_024 |
| *ubiA* | BVAF_024 | *ubiA* | Bfl025 | *ubiA* | BPEN_025 |
| *zur* | BVAF_025 | *zur* | Bfl026 | *zur* | BPEN_026 |
| *dnaB* | BVAF_026 | *dnaB* | Bfl027 | *dnaB* | BPEN_027 |
| *ssb* | BVAF_027 | *ssb* | Bfl028 | *ssb* | BPEN_028 |
| *yjcE* | BVAF_028 | *yjcE* | Bfl029 | *yjcE* | BPEN_029 |
| *gltP* | BVAF_029 | *gltP* | Bfl030 | *gltP* | BPEN_030 |
| *yjgF* | BVAF_030 | *yjgF* | Bfl031 | *yjgF* | BPEN_031 |
| *argI* | BVAF_031 | *argI* | Bfl032 | *argI* | BPEN_032 |
| *valS* | BVAF_032 | *valS* | Bfl033 | *valS* | BPEN_033 |
| *holC* | BVAF_033 | *holC* | Bfl034 | *holC* | BPEN_034 |
| *pepA* | BVAF_034 | *pepA* | Bfl035 | *pepA* | BPEN_035 |
| *yjgP* | BVAF_035 | *yjgP* | Bfl036 | *yjgP* | BPEN_036 |
| *yjgQ* | BVAF_036 | *yjgQ* | Bfl037 | *yjgQ* | BPEN_037 |
| *yidZ* | BVAF_037 | *yidZ* | Bfl038 | *yidZ* | BPEN_038 |
| tRNA-Leu | BVAF_038 | tRNA-Leu5 | Bfl039 | tRNA-Leu | BPEN_039 |
| BVAF_039 | BVAF_039 | identified in this study2 | | BPEN_040 | BPEN_040 |
| BVAF_040 | BVAF_040 | unknown | Bfl040 | *znuC* | BPEN_041 |
| BVAF_041 | BVAF_041 | unknown | Bfl041 | *znuB* | BPEN_042 |
| *yhbG* | BVAF_042 | *yhbG* | Bfl042 | *yhbG* | BPEN_043 |
| *lptA* | BVAF_043 | *yhbN* | Bfl043 | *yhbN* | BPEN_044 |
| *lptC* | BVAF_044 | *yrbK* | Bfl044 | *yrbK* | BPEN_045 |
| *yrbA* | BVAF_045 | *yrbA* | Bfl045 | *yrbA* | BPEN_046 |
| *murA* | BVAF_046 | *murA* | Bfl046 | *murA* | BPEN_047 |
| *degQ* | BVAF_047 | *degQ* | Bfl047 | *degQ* | BPEN_048 |
| *yhcB* | BVAF_048 | *yhcB* | Bfl048 | *yhcB* | BPEN_049 |
| *rplM* | BVAF_049 | *rplM* | Bfl049 | *rplM* | BPEN_050 |
| *rpsI* | BVAF_050 | *rpsI* | Bfl050 | *rpsI* | BPEN_051 |
| *yraP* | BVAF_051 | *yraP* | Bfl051 | *yraP* | BPEN_052 |
| *yraL* | BVAF_052 | *yraL* | Bfl052 | *yraL* | BPEN_053 |
| *rnpB* | BVAF_053 | *rnpB* | Bfl053 | *rnpB* | BPEN_054 |
| *yqjA* | BVAF_054 | *yqjA* | Bfl054 | *yqjA* | BPEN_055 |
| tRNA-Met | BVAF_055 | tRNA-Met3 | Bfl055 | tRNA-Met | BPEN_056 |
| *rpoD* | BVAF_056 | *rpoD* | Bfl056 | *rpoD* | BPEN_057 |
| *dnaG* | BVAF_057 | *dnaG* | Bfl057 | *dnaG* | BPEN_058 |
| *rpsU* | BVAF_058 | *rpsU* | Bfl058 | *rpsU* | BPEN_059 |
| *gcp* | BVAF_059 | *gcp* | Bfl059 | *gcp* | BPEN_060 |
| *ygiH* | BVAF_060 | *ygiH* | Bfl060 | *ygiH* | BPEN_061 |
| *folB* | BVAF_061 | *folB* | Bfl061 | *folB* | BPEN_062 |
|  |  |  |  | *bacA* | BPEN_063 |
| *cca* | BVAF_062 | *cca* | Bfl062 | *cca* | BPEN_064 |
| *hldE* | BVAF_063 | *rfaE* | Bfl063 | *hldE* | BPEN_065 |
| *yqiC* | BVAF_064 | *yqiC* | Bfl064 | *yqiC*  | BPEN_066 |
| *ribB* | BVAF_065 | *ribB* | Bfl065 | *ribB* | BPEN_067 |
| *plsC* | BVAF_066 | *plsC* | Bfl066 | *plsC* | BPEN_068 |
| *metC* | BVAF_067 | *metC* | Bfl067 | *metC* | BPEN_069 |
| tRNA-Phe | BVAF_068 | tRNA-Phe | Bfl068 | tRNA-Phe | BPEN_070 |
| *cutA* | BVAF_069 | *cutA* | Bfl069 | *cutA* | BPEN_071 |
| *groES* | BVAF_070 | *groES* | Bfl070 | *groES* | BPEN_072 |
| *groEL* | BVAF_071 | *groEL* | Bfl071 | *groEL* | BPEN_073 |
| *efp* | BVAF_072 | *efp* | Bfl072 | *efp* | BPEN_074 |
| *yjeP* | BVAF_073 | *yjeP* | Bfl073 | *yjeP* | BPEN_075 |
| *psd* | BVAF_074 | *psd* | Bfl074 | *psd* | BPEN_076 |
| *orn* | BVAF_075 | *orn* | Bfl075 | *orn* | BPEN_077 |
| tRNA-Gly | BVAF_076 | tRNA-Gly2 | Bfl076 | tRNA-Gly | BPEN_078 |
| *yjeE* | BVAF_077 | *yjeE* | Bfl077 | *yjeE* | BPEN_079 |
| *amiB* | BVAF_078 | *amiB* | Bfl078 | *amiB* | BPEN_080 |
| *miaA* | BVAF_079 | *miaA* | Bfl079 | *miaA* | BPEN_081 |
|  |  |  |  | *hfq* | BPEN_082 |
| *hflX* | BVAF_080 | *hflX* | Bfl080 | *hflX* | BPEN_083 |
| *hflK* | BVAF_081 | *hflK* | Bfl081 | *hflK* | BPEN_084 |
| *hflC* | BVAF_082 | *hflC* | Bfl082 | *hflC* | BPEN_085 |
| *purA* | BVAF_083 | *purA* | Bfl083 | *purA* | BPEN_086 |
| *rlmB* | BVAF_084 | *yjfH* | Bfl084 | *rlmB* | BPEN_087 |
| *rpsF* | BVAF_085 | *rpsF* | Bfl085 | *rpsF* | BPEN_088 |
| *rpsR* | BVAF_086 | *rpsR* | Bfl086 | *rpsR* | BPEN_089 |
| *rplI* | BVAF_087 | *rplI* | Bfl087 | *rplI* | BPEN_090 |
| *cysQ* | BVAF_088 | *cysQ* | Bfl088 | *cysQ* | BPEN_091 |
| *ytfM* | BVAF_089 | *ytfM* | Bfl089 | *ytfM* | BPEN_092 |
| *ytfN* | BVAF_090 | *ytfN* | Bfl090 | *ytfN* | BPEN_093 |
| *ppa* | BVAF_091 | *ppa* | Bfl091 | *ppa* | BPEN_094 |
| *ispB* | BVAF_092 | *ispB* | Bfl092 | *ispB* | BPEN_095 |
| *rplU* | BVAF_093 | *rplU* | Bfl093 | *rplU* | BPEN_096 |
| *rpmA* | BVAF_094 | *rpmA* | Bfl094 | *rpmA* | BPEN_097 |
| *obgE* | BVAF_095 | *yhbZ* | Bfl095 | *obgE* | BPEN_098 |
| *greA* | BVAF_096 | *greA* | Bfl096 | *greA* | BPEN_099 |
| *rlmE* | BVAF_097 | *ftsJ* | Bfl097 | *rrmJ* | BPEN_100 |
| *ftsH* | BVAF_098 | *ftsH* | Bfl098 | *hflB* | BPEN_101 |
| *folP* | BVAF_099 | *folP* | Bfl099 | *folP* | BPEN_102 |
| *glmM* | BVAF_100 | *glmM* | Bfl100 | *glmM* | BPEN_103 |
| *secG* | BVAF_101 | identified in this study2 | | *secG* | BPEN_104 |
| tRNA-Leu | BVAF_102 | tRNA-Leu4 | Bfl101 | tRNA-Leu | BPEN_105 |
| tRNA-Met | BVAF_103 | tRNA-Met2 | Bfl102 | tRNA-Met | BPEN_106 |
| *nusA* | BVAF_104 | *nusA* | Bfl103 | *nusA* | BPEN_107 |
| *infB* | BVAF_105 | *infB* | Bfl104 | *infB* | BPEN_108 |
| *rbfA* | BVAF_106 | *rbfA* | Bfl105 | *rbfA* | BPEN_109 |
| *truB* | BVAF_107 | *truB* | Bfl106 | *truB* | BPEN_110 |
| *rpsO* | BVAF_108 | *rpsO* | Bfl107 | *rpsO* | BPEN_111 |
| *pnp* | BVAF_109 | *pnp* | Bfl108 | *pnp* | BPEN_112 |
| *deaD* | BVAF_110 | *deaD* | Bfl109 | *deaD* | BPEN_113 |
| *holD* | BVAF_111 | *holD* | Bfl110 | *holD* | BPEN_114 |
| *thrA* | BVAF_112 | *thrA* | Bfl111 | *thrA* | BPEN_115 |
| *thrB* | BVAF_113 | *thrB* | Bfl112 | *thrB* | BPEN_116 |
| *thrC* | BVAF_114 | *thrC* | Bfl113 | *thrC* | BPEN_117 |
| *dnaK* | BVAF_115 | *dnaK* | Bfl114 | *dnaK* | BPEN_118 |
| *dnaJ* | BVAF_116 | *dnaJ* | Bfl115 | *dnaJ* | BPEN_119 |
| *rpsT* | BVAF_117 | *rpsT* | Bfl116 | *rpsT* | BPEN_120 |
| *ribF* | BVAF_118 | *ribF* | Bfl117 | *ribF* | BPEN_121 |
| *ileS* | BVAF_119 | *ileS* | Bfl118 | *ileS* | BPEN_122 |
| *lspA* | BVAF_120 | *lspA* | Bfl119 | *lspA* | BPEN_123 |
| *ispH* | BVAF_121 | *lytB*  | Bfl120 | *ispH* | BPEN_124 |
| *dapB* | BVAF_122 | *dapB* | Bfl121 | *dapB* | BPEN_125 |
| *carA* | BVAF_123 | *carA* | Bfl122 | *carA* | BPEN_126 |
| *carB* | BVAF_124 | *carB* | Bfl123 | *carB* | BPEN_127 |
| *folA* | BVAF_125 | *folA* | Bfl124 | *folA* | BPEN_128 |
| *apaH* | BVAF_126 | *apaH* | Bfl125 | *apaH* | BPEN_129 |
| *ksgA* | BVAF_127 | *ksgA* | Bfl126 | *ksgA* | BPEN_130 |
| *pdxA* | BVAF_128 | *pdxA* | Bfl127 | *pdxA* | BPEN_131 |
| *surA* | BVAF_129 | *surA* | Bfl128 | *surA* | BPEN_132 |
| *lptD* | BVAF_130 | *imp* | Bfl129 | *imp* | BPEN_133 |
| *leuD* | BVAF_131 | *leuD* | Bfl130 | *leuD* | BPEN_134 |
| *leuC* | BVAF_132 | *leuC* | Bfl131 | *leuC* | BPEN_135 |
| *leuB* | BVAF_133 | *leuB* | Bfl132 | *leuB* | BPEN_136 |
| *leuA* | BVAF_134 | *leuA* | Bfl133 | *leuA* | BPEN_137 |
| *mraW* | BVAF_135 | *mraW* | Bfl134 | *mraW* | BPEN_138 |
| *ftsL* | BVAF_136 | *ftsL* | Bfl135 | *ftsL* | BPEN_139 |
| *ftsI* | BVAF_137 | *ftsI* | Bfl136 | *ftsI* | BPEN_140 |
| *murE* | BVAF_138 | *murE* | Bfl137 | *murE* | BPEN_141 |
| *murF* | BVAF_139 | *murF* | Bfl138 | *murF* | BPEN_142 |
| *mraY* | BVAF_140 | *mraY* | Bfl139 | *mraY* | BPEN_143 |
| *murD* | BVAF_141 | *murD* | Bfl140 | *murD* | BPEN_144 |
| *ftsW* | BVAF_142 | *ftsW* | Bfl141 | *ftsW* | BPEN_145 |
| *murG* | BVAF_143 | *murG* | Bfl142 | *murG* | BPEN_146 |
| *murC* | BVAF_144 | *murC* | Bfl143 | *murC* | BPEN_147 |
| *ftsQ* | BVAF_145 | *ftsQ* | Bfl144 | *ftsQ* | BPEN_148 |
| *ftsA* | BVAF_146 | *ftsA* | Bfl145 | *ftsA* | BPEN_149 |
| *ftsZ* | BVAF_147 | *ftsZ* | Bfl146 | *ftsZ* | BPEN_150 |
| *lpxC* | BVAF_148 | *lpxC* | Bfl147 | *lpxC* | BPEN_151 |
| *secA* | BVAF_149 | *secA* | Bfl148 | *secA* | BPEN_152 |
|  |  |  |  | *coaE* | BPEN_153 |
| *dksA* | BVAF_150 | *dksA* | Bfl149 | *dksA* | BPEN_154 |
| *folK* | BVAF_151 | *folK* | Bfl150 | *folK* | BPEN_155 |
| *lpdA* | BVAF_152 | *lpdA* | Bfl151 | *lpdA* | BPEN_156 |
| *aceF* | BVAF_153 | *aceF* | Bfl152 | *aceF* | BPEN_157 |
| *aceE* | BVAF_154 | *aceE* | Bfl153 | *aceE* | BPEN_158 |
| *mrcB* | BVAF_155 | *mrcB* | Bfl154 | *mrcB* | BPEN_159 |
| *erpA* | BVAF_156 | *yadR* | Bfl155 | *yadR* | BPEN_160 |
| *pyrG* | BVAF_157 | *pyrG* | Bfl156 | *pyrG* | BPEN_161 |
| *eno* | BVAF_158 | *eno* | Bfl157 | *eno* | BPEN_162 |
| *cysJ* | BVAF_159 | *cysJ* | Bfl158 | *cysJ* | BPEN_163 |
| *cysI* | BVAF_160 | *cysI* | Bfl159 | *cysI* | BPEN_164 |
| *cysH* | BVAF_161 | *cysH* | Bfl160 | *cysH* | BPEN_165 |
| *cysG* | BVAF_162 | *cysG* | Bfl161 | *cysG* | BPEN_166 |
| *cysD* | BVAF_163 | *cysD* | Bfl162 | *cysD* | BPEN_167 |
| *cysN* | BVAF_164 | *cysN* | Bfl163 | *cysN* | BPEN_168 |
| *cysC* | BVAF_165 | *cysC* | Bfl164 | *cysC* | BPEN_169 |
| *ftsB* | BVAF_166 | *ygbQ* | Bfl165 | *ygbQ* | BPEN_170 |
| *ispD* | BVAF_167 | *ygbP*  | Bfl166 | *ispD* | BPEN_171 |
| *ispF* | BVAF_168 | *ispF*  |  | *ispF* | BPEN_172 |
| *nlpD* | BVAF_169 | *nlpD* | Bfl167 | *nlpD* | BPEN_173 |
| *alaS* | BVAF_170 | *alaS* | Bfl168 | *alaS* | BPEN_174 |
| *csrA* | BVAF_171 | *csrA* | Bfl169 | *csrA* | BPEN_175 |
| tRNA-Ser | BVAF_172 | tRNA-Ser4 | Bfl170 | tRNA-Ser | BPEN_176 |
| tRNA-Arg | BVAF_173 | tRNA-Arg4 | Bfl171 | tRNA-Arg | BPEN_177 |
|  |  | *ffh* | Bfl172 | *ffh* | BPEN_178 |
| *rpsP* | BVAF_174 | *rpsP* | Bfl173 | *rpsP* | BPEN_179 |
| *rimM* | BVAF_175 | *rimM* | Bfl174 | *rimM* | BPEN_180 |
| *trmD* | BVAF_176 | *trmD* | Bfl175 | *trmD* | BPEN_181 |
| *rplS* | BVAF_177 | *rplS* | Bfl176 | *rplS* | BPEN_182 |
| *aroF* | BVAF_178 | *aroF* | Bfl177 | *aroF* | BPEN_183 |
| *tyrA* | BVAF_179 | *tyrA* | Bfl178 | *tyrA* | BPEN_184 |
| *pheA* | BVAF_180 | *pheA* | Bfl179 | *pheA* | BPEN_185 |
| *yfiO* | BVAF_181 | *yfiO* | Bfl180 | *yfiO* | BPEN_186 |
| *rluD* | BVAF_182 | *rluD* | Bfl181 | *rluD* | BPEN_187 |
| *clpB* | BVAF_183 | *clpB* | Bfl182 | *clpB* | BPEN_188 |
| *murB* | BVAF_184 | *murB* | Bfl183 | *murB* | BPEN_189 |
| *birA* | BVAF_185 | *birA* | Bfl184 | *birA* | BPEN_190 |
|  |  |  |  | *coaA* | BPEN_191 |
| tRNA-Thr | BVAF_186 | tRNA-Thr3 | Bfl185 | tRNA-Thr | BPEN_192 |
| tRNA-Tyr | BVAF_187 | tRNA-Tyr | Bfl186 | tRNA-Tyr | BPEN_193 |
| tRNA-Gly | BVAF_188 | tRNA-Gly1 | Bfl187 | tRNA-Gly3 | BPEN_194 |
| tRNA-Thr | BVAF_189 | tRNA-Thr2 | Bfl188 | tRNA-Thr | BPEN_195 |
| *bfr* | BVAF_190 | *bfr* | Bfl189 | *bfr* | BPEN_196 |
| *rpsJ* | BVAF_191 | *rpsJ* | Bfl190 | *rpsJ* | BPEN_197 |
| *rplC* | BVAF_192 | *rplC* | Bfl191 | *rplC* | BPEN_198 |
| *rplD* | BVAF_193 | *rplD* | Bfl192 | *rplD* | BPEN_199 |
| *rplW* | BVAF_194 | *rplW* | Bfl193 | *rplW* | BPEN_200 |
| *rplB* | BVAF_195 | *rplB* | Bfl194 | *rplB* | BPEN_201 |
| *rpsS* | BVAF_196 | *rpsS* | Bfl195 | *rpsS* | BPEN_202 |
| *rplV* | BVAF_197 | *rplV* | Bfl196 | *rplV* | BPEN_203 |
| *rpsC* | BVAF_198 | *rpsC* | Bfl197 | *rpsC* | BPEN_204 |
| *rplP* | BVAF_199 | *rplP* | Bfl198 | *rplP* | BPEN_205 |
| *rpmC* | BVAF_200 | *rpmC* | Bfl199 | *rpmC* | BPEN_206 |
| *rpsQ* | BVAF_201 | *rpsQ* | Bfl200 | *rpsQ* | BPEN_207 |
| *rplN* | BVAF_202 | *rplN* | Bfl201 | *rplN* | BPEN_208 |
| *rplX* | BVAF_203 | *rplX* | Bfl202 | *rplX* | BPEN_209 |
| *rplE* | BVAF_204 | *rplE* | Bfl203 | *rplE* | BPEN_210 |
| *rpsN* | BVAF_205 | *rpsN* | Bfl204 | *rpsN* | BPEN_211 |
| *rpsH* | BVAF_206 | *rpsH* | Bfl205 | *rpsH* | BPEN_212 |
| *rplF* | BVAF_207 | *rplF* | Bfl206 | *rplF* | BPEN_213 |
| *rplR* | BVAF_208 | *rplR* | Bfl207 | *rplR* | BPEN_214 |
| *rpsE* | BVAF_209 | *rpsE* | Bfl208 | *rpsE* | BPEN_215 |
| *rpmD* | BVAF_210 | *rpmD* | Bfl209 | *rpmD*  | BPEN_216 |
| *rplO* | BVAF_211 | *rplO* | Bfl210 | *rplO* | BPEN_217 |
| *secY* | BVAF_212 | *secY* | Bfl211 | *secY* | BPEN_218 |
| *rpmJ* | BVAF_213 | *rpmJ* | Bfl212 | *rmpJ* | BPEN_219 |
| *rpsM* | BVAF_214 | *rpsM* | Bfl213 | *rpsM* | BPEN_220 |
| *rpsK* | BVAF_215 | *rpsK* | Bfl214 | *rpsK* | BPEN_221 |
| *rpsD* | BVAF_216 | *rpsD* | Bfl215 | *rpsD* | BPEN_222 |
| *rpoA* | BVAF_217 | *rpoA* | Bfl216 | *rpoA* | BPEN_223 |
| *rplQ* | BVAF_218 | *rplQ* | Bfl217 | *rplQ* | BPEN_224 |
| *fmt* | BVAF_219 | *fmt* | Bfl218 | *fmt* | BPEN_225 |
| *def* | BVAF_220 | *def* | Bfl219 | *def* | BPEN_226 |
| *yrdC* | BVAF_221 | *yrdC* | Bfl220 | *yrdC* | BPEN_227 |
| *aroE* | BVAF_222 | *aroE* | Bfl221 | *aroE* | BPEN_228 |
| 5S rRNA | BVAF_223 | 5S rRNA | Bfl222 | *rrf* | BPEN_229 |
| *gloB* | BVAF_224 | *gloB* | Bfl223 | *gloB* | BPEN_230 |
| *rnhA* | BVAF_225 | *rnhA* | Bfl224 | *rnhA* | BPEN_231 |
| *dnaQ* | BVAF_226 | *dnaQ* | Bfl225 | *dnaQ* | BPEN_232 |
| *gmhA* | BVAF_227 | *gmhA* | Bfl226 | *gmhA* | BPEN_233 |
| tRNA-Thr | BVAF_228 | tRNA-Thr1 | Bfl227 | tRNA-Thr | BPEN_234 |
| *ahpC* | BVAF_229 | *ahpC* | Bfl228 | *ahpC* | BPEN_235 |
| *queA*  | BVAF_230 | *queA* | Bfl229 |  |  |
| *tgt* | BVAF_231 | *tgt* | Bfl230 | *tgt* | BPEN_236 |
| *yajC* | BVAF_232 | *yajC* | Bfl231 | *yajC* | BPEN_237 |
| *secD* | BVAF_233 | *secD* | Bfl232 | *secD* | BPEN_238 |
| *secF* | BVAF_234 | *secF* | Bfl233 | *secF* | BPEN_239 |
| *ribD* | BVAF_235 | *ribD* | Bfl234 | *ribD* | BPEN_240 |
| *ribH* | BVAF_236 | *ribH* | Bfl235 | *ribH* | BPEN_241 |
| *nusB* | BVAF_237 | *nusB* | Bfl236 | *nusB* | BPEN_242 |
| *pgpA* | BVAF_238 | *pgpA* | Bfl237 | *pgpA* | BPEN_243 |
| *dxs* | BVAF_239 | *dxs* | Bfl238 | *dxs* | BPEN_244 |
|  |  |  |  | *ispA* | BPEN_245 |
| *thiI* | BVAF_240 | *thiI* | Bfl239 | *thiI* | BPEN_246 |
| *yajR* | BVAF_241 | *yajR* | Bfl240 | *yajR* | BPEN_247 |
| *cyoE* | BVAF_242 | *cyoE* | Bfl241 | *cyoE* | BPEN_248 |
| *cyoD* | BVAF_243 | *cyoD* | Bfl242 | *cyoD* | BPEN_249 |
| *cyoC* | BVAF_244 | *cyoC* | Bfl243 | *cyoC* | BPEN_250 |
| *cyoB* | BVAF_245 | *cyoB* | Bfl244 | *cyoB* | BPEN_251 |
| *cyoA* | BVAF_246 | *cyoA* | Bfl245 | *cyoA* | BPEN_252 |
| *clpP* | BVAF_247 | *clpP* | Bfl246 | *clpP* | BPEN_253 |
| *clpX* | BVAF_248 | *clpX* | Bfl247 | *clpX* | BPEN_254 |
| *yggX* | BVAF_249 | *yggX* | Bfl248 | *yggX* | BPEN_255 |
| *mutY* | BVAF_250 | *mutY* | Bfl249 | *mutY* | BPEN_256 |
| *yggS* | BVAF_251 |  |  |  |  |
| *yqgF* | BVAF_252 | *yqgF* | Bfl250 | *yqgF* | BPEN_257 |
| *yqgE* | BVAF_253 | *yqgE* | Bfl251 | *yqgE* | BPEN_258 |
| *metK* | BVAF_254 | *metK* | Bfl252 | *metK* | BPEN_259 |
| *speB* | BVAF_255 | *speB* | Bfl253 | *speB* | BPEN_260 |
| *pgk* | BVAF_256 | *pgk* | Bfl254 | *pgk* | BPEN_261 |
| *fbaA* | BVAF_257 | *fba* | Bfl255 | *fbaA* | BPEN_262 |
| *rpiA* | BVAF_258 | *rpiA* | Bfl256 | *rpiA* | BPEN_263 |
| *ygfA* | BVAF_259 | *ygfA* | Bfl257 | *ygfA* | BPEN_264 |
| *zapA* | BVAF_260 | *ygfE* | Bfl258 | *ygfE* | BPEN_265 |
| *ubiH* | BVAF_261 | *ubiH* | Bfl259 | *ubiH* | BPEN_266 |
| *visC* | BVAF_262 |  |  | *visC* | BPEN_267 |
| *ygfZ* | BVAF_263 | *ygfZ* | Bfl260 | *ygfZ* | BPEN_268 |
| *prfB* | BVAF_264 | *prfB* | Bfl261 | *prfB* | BPEN_269 |
| *lysS* | BVAF_265 | *lysS* | Bfl262 | *lysS* | BPEN_270 |
| *lysA* | BVAF_266 | *lysA* | Bfl263 | *lysA* | BPEN_271 |
| *rppH* | BVAF_267 | *ygdP* | Bfl264 | *ygdP* | BPEN_272 |
| *lgt* | BVAF_268 | *lgt* | Bfl265 | *lgt* | BPEN_273 |
| *recC* | BVAF_269 | *recC* | Bfl266 | *recC* | BPEN_274 |
| *recD* | BVAF_270 | *recD* | Bfl267 | *recD* | BPEN_275 |
| *recB* | BVAF_271 | *recB* | Bfl268 | *recB* | BPEN_276 |
| *dapD* | BVAF_272 | *dapD* | Bfl269 | *dapD* | BPEN_277 |
| *map* | BVAF_273 | *map* | Bfl270 | *map* | BPEN_278 |
| *rpsB* | BVAF_274 | *rpsB* | Bfl271 | *rpsB* | BPEN_279 |
| *tsf* | BVAF_275 | *tsf* | Bfl272 | *tsf* | BPEN_280 |
| *pyrH* | BVAF_276 | *pyrH* | Bfl273 | *pyrH* | BPEN_281 |
| *frr* | BVAF_277 | *frr* | Bfl274 | *frr* | BPEN_282 |
| *dxr* | BVAF_278 | *dxr* | Bfl275 | *dxr* | BPEN_283 |
| *uppS* | BVAF_279 | *uppS* | Bfl276 | *uppS* | BPEN_284 |
| *cdsA* | BVAF_280 | *cdsA* | Bfl277 | *cdsA* | BPEN_285 |
| *rseP* | BVAF_281 | *yaeL* | Bfl278 | *ecfE* | BPEN_286 |
| *yaeT* | BVAF_282 | *yaeT* | Bfl279 | *yaeT* | BPEN_287 |
| *skp* | BVAF_283 | *hlpA* | Bfl280 | *hlpA* | BPEN_288 |
| *lpxD* | BVAF_284 | *lpxD* | Bfl281 | *lpxD* | BPEN_289 |
| *fabZ* | BVAF_285 | *fabZ* | Bfl282 | *fabZ* | BPEN_290 |
| *lpxA* | BVAF_286 | *lpxA* | Bfl283 | *lpxA* | BPEN_291 |
| *lpxB* | BVAF_287 | *lpxB* | Bfl284 | *lpxB* | BPEN_292 |
| *rnhB* | BVAF_288 | *rnhB* | Bfl285 | *rnhB* | BPEN_293 |
| *dnaE* | BVAF_289 | *dnaE* | Bfl286 | *dnaE* | BPEN_294 |
| *accA* | BVAF_290 | *accA* | Bfl287 | *accA* | BPEN_295 |
| *tilS* | BVAF_291 | *mesJ* | Bfl288 | *tilS* | BPEN_296 |
| *proS* | BVAF_292 | *proS* | Bfl289 | *proS* | BPEN_297 |
| *gmhB* | BVAF_293 | *yaeD* | Bfl290 | *gmhB* | BPEN_298 |
| *accC* | BVAF_294 | *accC* | Bfl291 | *accC* | BPEN_299 |
| *accB* | BVAF_295 | *accB* | Bfl292 | *accB* | BPEN_300 |
| *aroQ* | BVAF_296 | *aroQ* | Bfl293 | *aroQ* | BPEN_301 |
| *mreB* | BVAF_297 | *mreB* | Bfl294 | *mreB* | BPEN_302 |
| *mreC* | BVAF_298 | *mreC* | Bfl295 | *mreC* | BPEN_303 |
| *mreD* | BVAF_299 | *mreD* | Bfl296 | *mreD* | BPEN_304 |
| *tldD* | BVAF_300 | *tldD* | Bfl297 | *tldD* | BPEN_305 |
| *pmbA* | BVAF_301 | *pmbA* | Bfl298 | *pmbA* | BPEN_306 |
| *lon* | BVAF_302 | *lon* | Bfl299 | *lon* | BPEN_307 |
|  |  | *ffs*4 |  | *ffs*4 |  |
| *apt* | BVAF_303 | *apt* | Bfl300 | *apt* | BPEN_308 |
| *dnaX* | BVAF_304 | *dnaX* | Bfl301 | *dnaX* | BPEN_309 |
| *adk* | BVAF_305 | *adk* | Bfl302 | *adk* | BPEN_310 |
| *lpxH* | BVAF_306 | *ybbF* | Bfl303 | *lpxH* | BPEN_311 |
| *cysS* | BVAF_307 | *cysS* | Bfl304 | *cysS* | BPEN_312 |
| *folD* | BVAF_308 | *folD* | Bfl305 | *folD* | BPEN_313 |
| tRNA-Arg | BVAF_309 | tRNA-Arg3 | Bfl306 | tRNA-Arg | BPEN_314 |
| *ytfF* | BVAF_310 | *ytfF* | Bfl307 | *ytfF* | BPEN_315 |
| *mrdB* | BVAF_311 | *mrdB* | Bfl308 | *mrdB* | BPEN_316 |
| *mrdA* | BVAF_312 | *mrdA* | Bfl309 | *mrdA* | BPEN_317 |
| *ybeB* | BVAF_313 | *ybeB* | Bfl310 | *ybeB* | BPEN_318 |
| *holA* | BVAF_314 | *holA* | Bfl311 | *holA* | BPEN_319 |
| *rlpB* | BVAF_315 | *rlpB* | Bfl312 | *rlpB* | BPEN_320 |
| *leuS* | BVAF_316 | *leuS* | Bfl313 | *leuS* | BPEN_321 |
| *lnt* | BVAF_317 | *lnt* | Bfl314 | *lnt* | BPEN_322 |
| *ybeX* | BVAF_318 | *ybeX* | Bfl315 | *ybeX* | BPEN_323 |
| *ybeY* | BVAF_319 | *ybeY* | Bfl316 | *ybeY* | BPEN_324 |
| *ybeZ* | BVAF_320 | *phoL* | Bfl317 | *phoL* | BPEN_325 |
| *ubiF* | BVAF_321 | *ubiF* | Bfl318 | *ubiF* | BPEN_326 |
| tRNA-Gln | BVAF_322 | tRNA-Gln | Bfl319 | tRNA-Gln | BPEN_327 |
| tRNA-Leu | BVAF_323 | tRNA-Leu3 | Bfl320 | tRNA-Leu | BPEN_328 |
| tRNA-Met | BVAF_324 | tRNA-Met1 | Bfl321 | tRNA-Met | BPEN_329 |
| *nagA* | BVAF_325 | *nagA* | Bfl322 | *nagA* | BPEN_330 |
| *nagB* | BVAF_326 | *nagB* | Bfl323 | *nagB* | BPEN_331 |
| *glnS* | BVAF_327 | *glnS* | Bfl324 | *glnS* | BPEN_332 |
| *fldA* | BVAF_328 | *fldA* | Bfl325 | *fldA* | BPEN_333 |
|  |  |  |  | *ybfF* | BPEN_334 |
|  |  | *pgm* | Bfl326 | *pgm* | BPEN_335 |
|  |  |  |  | BPEN_336 | BPEN_336 |
| *sdhC* | BVAF_329 | *sdhC* | Bfl327 | *sdhC* | BPEN_337 |
| *sdhD* | BVAF_330 | *sdhD* | Bfl328 | *sdhD* | BPEN_338 |
| *sdhA* | BVAF_331 | *sdhA* | Bfl329 | *sdhA* | BPEN_339 |
| *sdhB* | BVAF_332 | *sdhB* | Bfl330 | *sdhB* | BPEN_340 |
| *sucA* | BVAF_333 | *sucA* | Bfl331 | *sucA* | BPEN_341 |
| *sucB* | BVAF_334 | *sucB* | Bfl332 | *sucB* | BPEN_342 |
| *sucC* | BVAF_335 | *sucC* | Bfl333 | *sucC* | BPEN_343 |
| *sucD* | BVAF_336 | *sucD* | Bfl334 | *sucD* | BPEN_344 |
| *tolQ* | BVAF_337 | *tolQ* | Bfl335 | *tolQ* | BPEN_345 |
| *tolR* | BVAF_338 | *tolR* | Bfl336 | *tolR* | BPEN_346 |
| *tolA* | BVAF_339 | *tolA* | Bfl337 | *tolA* | BPEN_347 |
| *tolB* | BVAF_340 | *tolB* | Bfl338 | *tolB* | BPEN_348 |
| *pal* | BVAF_341 | *pal* | Bfl339 | *pal* | BPEN_349 |
| *ygbF* | BVAF_342 | *ybgF* | Bfl340 | *ybgF* | BPEN_350 |
| *pgl* | BVAF_343 | *ybhE* | Bfl341 | *ybhE* | BPEN_351 |
| *gpmA* | BVAF_344 | *gpmA* | Bfl342 | *gpmA* | BPEN_352 |
| *ybhL* | BVAF_345 | *ybhL* | Bfl343 | *ybhL* | BPEN_353 |
| *engD* | BVAF_346 | *ychF* | Bfl344 | *ychF* | BPEN_354 |
| *pth* | BVAF_347 | *pth* | Bfl345 | *pth* | BPEN_355 |
| *prs* | BVAF_348 | *prsA* | Bfl346 | *prsA* | BPEN_356 |
| *ispE* | BVAF_349 | *ipk* | Bfl347 | *ispE* | BPEN_357 |
| *lolB* | BVAF_350 |  |  | *lolB* | BPEN_358 |
| *prfA* | BVAF_351 | *prfA* | Bfl348 | *prfA* | BPEN_359 |
| *hemK* | BVAF_352 | *hemK* | Bfl349 | *hemK* | BPEN_360 |
| *kdsA* | BVAF_353 | *kdsA* | Bfl350 | *kdsA* | BPEN_361 |
| *thrS* | BVAF_354 | *thrS* | Bfl351 | *thrS* | BPEN_362 |
| *infC* | BVAF_355 | *infC* | Bfl352 | *infC* | BPEN_363 |
| *rpmI* | BVAF_356 | *rpmI* | Bfl353 | *rpmI* | BPEN_364 |
| *rplT* | BVAF_357 | *rplT* | Bfl354 | *rplT* | BPEN_365 |
| *pheS* | BVAF_358 | *pheS* | Bfl355 | *pheS* | BPEN_366 |
| *pheT* | BVAF_359 | *pheT* | Bfl356 | *pheT* | BPEN_367 |
| *lplA* | BVAF_360 | *lplA* | Bfl357 | *lplA* | BPEN_368 |
| *sufA* | BVAF_361 | *sufA* | Bfl358 | *sufA* | BPEN_369 |
| *sufB* | BVAF_362 | *sufB* | Bfl359 | *sufB* | BPEN_370 |
| *sufC* | BVAF_363 | *sufC* | Bfl360 | *sufC* | BPEN_371 |
| *sufD* | BVAF_364 | *sufD* | Bfl361 | *sufD* | BPEN_372 |
| *sufS* | BVAF_365 | *sufS* | Bfl362 | *sufS* | BPEN_373 |
| *sufE* | BVAF_366 | *ynhA* | Bfl363 | *sufE* | BPEN_374 |
| *lpp* | BVAF_367 | *lpp* | Bfl364 | *lpp* | BPEN_375 |
| tRNA-Val | BVAF_368 | tRNA-Val2 | Bfl365 | tRNA-Val | BPEN_376 |
| *ribE* | BVAF_369 | *ribE* | Bfl366 | *ribE* | BPEN_377 |
| *grxD* | BVAF_370 | *ydhD* | Bfl367 | *ydhD* | BPEN_378 |
| *rnt* | BVAF_371 | *rnt* | Bfl368 | *rnt* | BPEN_379 |
| *slyA* | BVAF_372 | *slyA* | Bfl369 | *slyA* | BPEN_380 |
| *pdxH* | BVAF_373 | *pdxH* | Bfl370 | *pdxH* | BPEN_381 |
| *tyrS* | BVAF_374 | *tyrS* | Bfl371 | *tyrS* | BPEN_382 |
| *nth* | BVAF_375 | *nth* | Bfl372 | *nth* | BPEN_383 |
| *fumC* | BVAF_376 | *fumC* | Bfl373 | *fumC* | BPEN_384 |
| BVAF_377 | BVAF_377 | *eaeH* | Bfl374 | *eaeH* | BPEN_385 |
| *ubiX* | BVAF_378 | *ubiX* | Bfl375 | *ubiX* | BPEN_386 |
| *kdsB* | BVAF_379 | *kdsB* | Bfl376 | *kdsB* | BPEN_387 |
| *ycaR* | BVAF_380 | *ycaR* | Bfl377 | *ycaR* | BPEN_388 |
| *lpxK* | BVAF_381 | *lpxK* | Bfl378 | *lpxK* | BPEN_389 |
| *msbA* | BVAF_382 | *msbA* | Bfl379 | *msbA* | BPEN_390 |
| *rpsA* | BVAF_383 | *rpsA* | Bfl380 | *rpsA* | BPEN_391 |
| *cmk* | BVAF_384 | *cmk* | Bfl381 | *cmk* | BPEN_392 |
| *aroA* | BVAF_385 | *aroA* | Bfl382 | *aroA* | BPEN_393 |
| *serC* | BVAF_386 | *serC* | Bfl383 | *serC* | BPEN_394 |
| *serS* | BVAF_387 | *serS* | Bfl384 | *serS* | BPEN_395 |
| *lolA* | BVAF_388 | *lolA* | Bfl385 | *lolA* | BPEN_396 |
| *ftsK* | BVAF_389 | *ftsK* | Bfl386 | *ftsK* | BPEN_397 |
| *trxB* | BVAF_390 | *trxB* | Bfl387 | *trxB* | BPEN_398 |
| *infA* | BVAF_391 | *infA* | Bfl388 | *infA* | BPEN_399 |
| *oprC* | BVAF_392 | *oprC* | Bfl389 | *oprC* | BPEN_400 |
| *yqeI* | BVAF_393 | *yqeI* | Bfl390 | *yqeI* | BPEN_401 |
| *emtA* | BVAF_394 | *mltE* | Bfl391 | *emtA* | BPEN_402 |
|  |  |  |  | *mntR* | BPEN_403 |
| *mnmA* | BVAF_395 | *mnmA* | Bfl392 | *mnmA* | BPEN_404 |
| *purB* | BVAF_396 | *purB* | Bfl393 | *purB* | BPEN_405 |
| *lolE* | BVAF_397 | *lolE* | Bfl394 | *lolE* | BPEN_406 |
| *lolD* | BVAF_398 | *lolD* | Bfl395 | *lolD* | BPEN_407 |
| *lolC* | BVAF_399 | *ycfU* | Bfl396 | *lolC* | BPEN_408 |
| *ycfM* | BVAF_400 | *ycfM* | Bfl397 | *ycfM* | BPEN_409 |
| *hinT* | BVAF_401 | *ycfF* | Bfl398 | *ycfF* | BPEN_410 |
| *ycfH* | BVAF_402 | *ycfH* | Bfl399 | *ycfH* | BPEN_411 |
| *holB* | BVAF_403 | *holB* | Bfl400 | *holB* | BPEN_412 |
| *tmk* | BVAF_404 | *tmk* | Bfl401 | *tmk* | BPEN_413 |
| *pabC* | BVAF_405 | *pabC* | Bfl402 | *pabC* | BPEN_414 |
| *acpP* | BVAF_406 | *acpP* | Bfl403 | *acpP* | BPEN_415 |
| *fabG* | BVAF_407 | *fabG* | Bfl404 | *fabG* | BPEN_416 |
| *fabD* | BVAF_408 | *fabD* | Bfl405 | *fabD* | BPEN_417 |
| *fabH* | BVAF_409 | *fabH* | Bfl406 | *fabH* | BPEN_418 |
| *plsX* | BVAF_410 | *plsX* | Bfl407 | *plsX* | BPEN_419 |
| *rpmF* | BVAF_411 | *rpmF* | Bfl408 | *rpmF* | BPEN_420 |
|  |  | *rluC* | Bfl409 | *rluC* | BPEN_421 |
| *rne* | BVAF_412 | *rne* | Bfl410 | *rne* | BPEN_422 |
|  |  | *htrB* | Bfl411 | *lpxL* | BPEN_423 |
| *erfK* | BVAF_413 | *ybiS* | Bfl412 | *ybiS* | BPEN_424 |
| tRNA-Leu | BVAF_414 | tRNA-Leu2 | Bfl413 | tRNA-Leu | BPEN_425 |
| tRNA-Cys | BVAF_415 | tRNA-Cys | Bfl414 | tRNA-Cys | BPEN_426 |
| *pgsA* | BVAF_416 | *pgsA* | Bfl415 | *pgsA* | BPEN_427 |
| tRNA-Lys | BVAF_417 | tRNA-Lys2 | Bfl416 | tRNA-Lys | BPEN_428 |
| tRNA-Ser | BVAF_418 | tRNA-Ser3 | Bfl417 | tRNA-Ser | BPEN_429 |
| *tusE* | BVAF_419 | *yccK* | Bfl418 | *yccK* | BPEN_430 |
| *hspQ* | BVAF_420 | *yccV* | Bfl419 | *yccV* | BPEN_431 |
| *fabA* | BVAF_421 | *fabA* | Bfl420 | *fabA* | BPEN_432 |
| *asnS* | BVAF_422 | *asnC* | Bfl421 | *asnC* | BPEN_433 |
| *aspC* | BVAF_423 | *aspC* | Bfl422 | *aspC* | BPEN_434 |
| *ycbL* | BVAF_424 | *ycbL* | Bfl423 | *ycbL* | BPEN_435 |
| *fabI* | BVAF_425 | *fabI* | Bfl424 | *fabI* | BPEN_436 |
| *ribA* | BVAF_426 | *ribA* | Bfl425 | *ribA* | BPEN_437 |
| *trpE* | BVAF_427 | *trpE* | Bfl426 | *trpE* | BPEN_438 |
| *trpG* | BVAF_428 | *trpG* | Bfl427 | *trpG* | BPEN_439 |
| *trpD* | BVAF_429 | *trpD* | Bfl428 | *trpD* | BPEN_440 |
| *trpC* | BVAF_430 | *trpC* | Bfl429 | *trpC* | BPEN_441 |
| *trpB* | BVAF_431 | *trpB* | Bfl430 | *trpB* | BPEN_442 |
| *trpA* | BVAF_432 | *trpA* | Bfl431 | *trpA* | BPEN_443 |
|  |  |  |  | *yciC* | BPEN_444 |
| *tonB* | BVAF_433 | *tonB* | Bfl432 | *tonB* | BPEN_445 |
| *cls* | BVAF_434 | *cls* | Bfl433 | *cls* | BPEN_446 |
| *tdk* | BVAF_435 | *tdk* | Bfl434 | *tdk* | BPEN_447 |
|  |  |  |  | *hns* | BPEN_448 |
| *xthA* | BVAF_436 | *xthA* | Bfl435 | *xthA* | BPEN_449 |
| *sppA* | BVAF_437 | *sppA* | Bfl436 | *sppA* | BPEN_450 |
| *gap* | BVAF_438 | *gapA* | Bfl437 | *gapA* | BPEN_451 |
| *dsbB* | BVAF_439 | *dsbB* | Bfl438 | *dsbB* | BPEN_452 |
| *minC* | BVAF_440 | *minC* | Bfl439 | *minC* | BPEN_453 |
| *minD* | BVAF_441 | *minD* | Bfl440 | *minD* | BPEN_454 |
| *minE* | BVAF_442 | *minE* | Bfl441 | *minE* | BPEN_455 |
| *yeaZ* | BVAF_443 | *yeaZ* | Bfl442 | *yeaZ* | BPEN_456 |
| *pabB* | BVAF_444 | *pabB* | Bfl443 | *pabB* | BPEN_457 |
| *yoaE* | BVAF_445 | *yoaE* | Bfl444 | *yoaE* | BPEN_458 |
| *manX* | BVAF_446 | *manX* | Bfl445 | *manX* | BPEN_459 |
| *manY* | BVAF_447 | *manY* | Bfl446 | *manY* | BPEN_460 |
| *manZ* | BVAF_448 | *manZ* | Bfl447 | *manZ* | BPEN_461 |
| *cspC* | BVAF_449 | *cspC* | Bfl448 | *cspC* | BPEN_462 |
|  |  |  |  | *htpX* | BPEN_463 |
| *zwf* | BVAF_450 | *zwf* | Bfl449 | *zwf* | BPEN_464 |
| *pykA* | BVAF_451 | *pykA* | Bfl450 | *pykA* | BPEN_465 |
| *yebA* | BVAF_452 | *yebA* | Bfl451 | *yebA* | BPEN_466 |
| *aspS* | BVAF_453 | *aspS* | Bfl452 | *aspS* | BPEN_467 |
| *argS* | BVAF_454 | *argS* | Bfl453 | *argS* | BPEN_468 |
| *mviN* | BVAF_455 | *mviN* | Bfl454 | *mviN* | BPEN_469 |
| *mdtH* | BVAF_456 | *yceL* | Bfl455 | *yceL* | BPEN_470 |
| tRNA-Ser | BVAF_457 | tRNA-Ser2 | Bfl456 | tRNA-Ser | BPEN_471 |
| tRNA-Ser | BVAF_458 | tRNA-Ser1 | Bfl457 | tRNA-Ser | BPEN_472 |
| tRNA-Asn | BVAF_459 | tRNA-Asn | Bfl458 | tRNA-Asn | BPEN_473 |
| *gutQ* | BVAF_460 | *gutQ* | Bfl459 | *gutQ* | BPEN_474 |
| *yeeX* | BVAF_461 | *yeeX* | Bfl460 | *yeeX* | BPEN_475 |
| *sbcB* | BVAF_462 | *sbcB* | Bfl461 | *sbcB* | BPEN_476 |
| *hisG* | BVAF_463 | *hisG* | Bfl462 | *hisG* | BPEN_477 |
| *hisD* | BVAF_464 | *hisD* | Bfl463 | *hisD* | BPEN_478 |
| *hisC* | BVAF_465 | *hisC* | Bfl464 | *hisC* | BPEN_479 |
| *hisB* | BVAF_466 | *hisB* | Bfl465 | *hisB* | BPEN_480 |
| *hisH* | BVAF_467 | *hisH* | Bfl466 | *hisH* | BPEN_481 |
| *hisA* | BVAF_468 | *hisA* | Bfl467 | *hisA* | BPEN_482 |
| *hisF* | BVAF_469 | *hisF* | Bfl468 | *hisF* | BPEN_483 |
| *hisI* | BVAF_470 | *hisI* | Bfl469 | *hisI* | BPEN_484 |
| *gnd* | BVAF_471 | *gnd* | Bfl470 | *gnd* | BPEN_485 |
| *metG* | BVAF_472 | *metG* | Bfl471 | *metG* | BPEN_486 |
| *folE* | BVAF_473 | *folE* | Bfl472 | *folE* | BPEN_487 |
| *rplY* | BVAF_474 | *rplY* | Bfl473 | *rplY* | BPEN_488 |
|  |  |  |  | tRNA-Pro | BPEN_489 |
| *ddl* | BVAF_475 | *ddl* | Bfl474 | *ddl* | BPEN_490 |
| *omp* | BVAF_476 | *omp* | Bfl475 | *omp* | BPEN_491 |
| *gyrA* | BVAF_477 | *gyrA* | Bfl476 | *gyrA* | BPEN_492 |
| *ubiG* | BVAF_478 | *ubiG* | Bfl477 | *ubiG* | BPEN_493 |
| *nrdA* | BVAF_479 | *nrdA* | Bfl478 | *nrdA* | BPEN_494 |
| *nrdB* | BVAF_480 | *nrdB* | Bfl479 | *nrdB* | BPEN_495 |
| *yfaE* | BVAF_481 | *yfaE* | Bfl480 | *yfaE* | BPEN_496 |
| *nuoN* | BVAF_482 | *nuoN* | Bfl481 | *nuoN* | BPEN_497 |
| *nuoM* | BVAF_483 | *nuoM* | Bfl482 | *nuoM* | BPEN_498 |
| *nuoL* | BVAF_484 | *nuoL* | Bfl483 | *nuoL* | BPEN_499 |
| *nuoK* | BVAF_485 | *nuoK* | Bfl484 | *nuoK* | BPEN_500 |
| *nuoJ* | BVAF_486 | *nuoJ* | Bfl485 | *nuoJ* | BPEN_501 |
| *nuoI* | BVAF_487 | *nuoI* | Bfl486 | *nuoI* | BPEN_502 |
| *nuoH* | BVAF_488 | *nuoH* | Bfl487 | *nuoH* | BPEN_503 |
| *nuoG* | BVAF_489 | *nuoG* | Bfl488 | *nuoG* | BPEN_504 |
| *nuoF* | BVAF_490 | *nuoF* | Bfl489 | *nuoF* | BPEN_505 |
| *nuoE* | BVAF_491 | *nuoE* | Bfl490 | *nuoE* | BPEN_506 |
| *nuoC* | BVAF_492 | *nuoCD* | Bfl491 | *nuoCD* | BPEN_507 |
| *nuoB* | BVAF_493 | *nuoB* | Bfl492 | *nuoB* | BPEN_508 |
| *nuoA* | BVAF_494 | *nuoA* | Bfl493 | *nuoA* | BPEN_509 |
| *folC* | BVAF_495 | *folC* | Bfl494 | *folC* | BPEN_510 |
| *accD* | BVAF_496 | *accD* | Bfl495 | *accD* | BPEN_511 |
| *truA* | BVAF_497 | *truA* | Bfl496 | *truA* | BPEN_512 |
| *pdxB* | BVAF_498 | *pdxB* | Bfl497 | *pdxB* | BPEN_513 |
| *fabB* | BVAF_499 | *fabB* | Bfl498 | *fabB* | BPEN_514 |
| *yfcB* | BVAF_500 | *yfcB* | Bfl499 | *yfcB* | BPEN_515 |
| *aroC* | BVAF_501 | *aroC* | Bfl500 | *aroC* | BPEN_516 |
| tRNA-Arg | BVAF_502 | tRNA-Arg-2 | Bfl501 | tRNA-Arg | BPEN_517 |
| *mntH* | BVAF_503 | *mntH* | Bfl502 | *mntH* | BPEN_518 |
|  |  | *nupC* | Bfl503 | *nupC* | BPEN_519 |
|  |  |  |  | tRNA-Ala | BPEN_520 |
| *gltX* | BVAF_504 | *gltX* | Bfl504 | *gltX* | BPEN_521 |
| tRNA-Val | BVAF_505 | tRNA-Val1 | Bfl505 | tRNA-Val | BPEN_522 |
| tRNA-Lys | BVAF_506 | tRNA-Lys1 | Bfl506 | tRNA-Lys | BPEN_523 |
| *ligA* | BVAF_507 | *lig* | Bfl507 | *ligA* | BPEN_524 |
| *cysK* | BVAF_508 | *cysK* | Bfl508 | *cysK* | BPEN_525 |
| *ptsH* | BVAF_509 | *ptsH* | Bfl509 | *ptsH* | BPEN_526 |
| *ptsI* | BVAF_510 | *ptsI* | Bfl510 | *ptsI* | BPEN_527 |
| *cysA* | BVAF_511 | *cysA* | Bfl511 | *cysA* | BPEN_528 |
| *cysW* | BVAF_512 | *cysW* | Bfl512 | *cysW* | BPEN_529 |
| *cysU* | BVAF_513 | *cysU* | Bfl513 | *cysU* | BPEN_530 |
| *cysP* | BVAF_514 | *cysP* | Bfl514 | *cysP* | BPEN_531 |
| *tal* | BVAF_515 | *talA* | Bfl515 | *talA* | BPEN_532 |
| *tktA* | BVAF_516 | *tktA* | Bfl516 | *tktA* | BPEN_533 |
| *dapE* | BVAF_517 | *dapE* | Bfl517 | *dapE* | BPEN_534 |
|  |  | identified in this study2 | | BPEN_535 | BPEN_535 |
| *dapA* | BVAF_518 | *dapA* | Bfl518 | *dapA* | BPEN_536 |
| *bcp*  | BVAF_519 | *bcp* | Bfl519 | *bcp* | BPEN_537 |
| *upp* | BVAF_520 | *upp* | Bfl520 | *upp* | BPEN_538 |
| BVAF_521 | BVAF_521 | identified in this study2 | | BPEN_539 | BPEN_539 |
| *ureG* | BVAF_522 | *ureG* | Bfl521 | *ureG* | BPEN_540 |
| *ureF* | BVAF_523 | *ureF* | Bfl522 | *ureF* | BPEN_541 |
| *ureC* | BVAF_524 | *ureC* | Bfl523 | *ureC* | BPEN_542 |
| *ureB* | BVAF_525 | *ureB* | Bfl524 | *ureB* | BPEN_543 |
| *ureA* | BVAF_526 | *ureA* | Bfl525 | *ureA* | BPEN_544 |
| *ureD* | BVAF_527 | *ureD* | Bfl526 | *ureD* | BPEN_545 |
| *guaA* | BVAF_528 | *guaA* | Bfl527 | *guaA* | BPEN_546 |
| *guaB* | BVAF_529 | *guaB* | Bfl528 | *guaB* | BPEN_547 |
| *mqo* | BVAF_530 | *mqo* | Bfl529 | *mqo* | BPEN_548 |
| *engA* | BVAF_531 | *engA* | Bfl530 | *engA* | BPEN_549 |
| *hisS* | BVAF_532 | *hisS* | Bfl531 | *hisS* | BPEN_550 |
| *ispG* | BVAF_533 | *gcpE*  | Bfl532 | *ispG* | BPEN_551 |
| *rlmN* | BVAF_534 |  |  | *yfgB* | BPEN_552 |
| *ndk* | BVAF_535 | *ndk* | Bfl533 | *ndk* | BPEN_553 |
| *iscS* | BVAF_536 | *nifS* | Bfl534 | *nifS* | BPEN_554 |
| *suhB* | BVAF_537 | *suhB* | Bfl535 | *suhB* | BPEN_555 |
| *glyA* | BVAF_538 | *glyA* | Bfl536 | *glyA* | BPEN_556 |
| *tadA* | BVAF_539 | *yfhC* | Bfl537 | *tadA* | BPEN_557 |
| *acpS* | BVAF_540 | *acpS* | Bfl538 | *acpS* | BPEN_558 |
| *pdxJ* | BVAF_541 | *pdxJ* | Bfl539 | *pdxJ* | BPEN_559 |
| *rnc* | BVAF_542 | *rnc* | Bfl540 | *rnc* | BPEN_560 |
| *lepB* | BVAF_543 | *lepB* | Bfl541 | *lepB* | BPEN_561 |
| *lepA* | BVAF_544 | *lepA* | Bfl542 | *lepA* | BPEN_562 |
| *ung* | BVAF_545 | *ung* | Bfl543 | *ung* | BPEN_563 |
| *grpE* | BVAF_546 | *grpE* | Bfl544 | *grpE* | BPEN_564 |
| *ppnK* | BVAF_547 | *ppnK* | Bfl545 | *ppnK* | BPEN_565 |
| *smpA* | BVAF_548 | *smpA* | Bfl546 | *smpA* | BPEN_566 |
| *yfjG* | BVAF_549 | *yfjG* | Bfl547 | *yfjG* | BPEN_567 |
| *smpB* | BVAF_550 | *smpB* | Bfl548 | *smpB* | BPEN_568 |
| tmRNA | BVAF_551 | tmRNA | Bfl549 | tmRNA | BPEN_569 |
| *qacE* | BVAF_552 | *emrE* | Bfl550 | *emrE* | BPEN_570 |
| *pssA* | BVAF_553 | *pssA* | Bfl551 | *pssA* | BPEN_571 |
| 23s rRNA | BVAF_554 | 23S rRNA | Bfl552 | *rrl* | BPEN_572 |
| tRNA-Glu | BVAF_555 | tRNA-Glu | Bfl553 | tRNA-Glu | BPEN_573 |
| 16s rRNA | BVAF_556 | 16S rRNA | Bfl554 | *rrs* | BPEN_574 |
| *purH* | BVAF_557 | *purH* | Bfl555 | *purH* | BPEN_575 |
| *rpoC* | BVAF_558 | *rpoC* | Bfl556 | *rpoC* | BPEN_576 |
| *rpoB* | BVAF_559 | *rpoB* | Bfl557 | *rpoB* | BPEN_577 |
| *rplL* | BVAF_560 | *rplL* | Bfl558 | *rplL* | BPEN_578 |
| *rplJ* | BVAF_561 | *rplJ* | Bfl559 | *rplJ* | BPEN_579 |
| *rplA* | BVAF_562 | *rplA* | Bfl560 | *rplA* | BPEN_580 |
| *rplK* | BVAF_563 | *rplK* | Bfl561 | *rplK* | BPEN_581 |
| *nusG* | BVAF_564 | *nusG* | Bfl562 | *nusG* | BPEN_582 |
| *secE* | BVAF_565 | *secE* | Bfl563 | *secE* | BPEN_583 |
| *tuf* | BVAF_566 | *tuf* | Bfl564 | *tuf* | BPEN_584 |
| *fusA* | BVAF_567 | *fusA* | Bfl565 | *fusA* | BPEN_585 |
| *rpsG* | BVAF_568 | *rpsG* | Bfl566 | *rpsG* | BPEN_586 |
| *rpsL* | BVAF_569 | *rpsL* | Bfl567 | *rpsL* | BPEN_587 |
| *pabA* | BVAF_570 | *pabA* | Bfl568 | *pabA* | BPEN_588 |
| *trpS* | BVAF_571 | *trpS* | Bfl569 | *trpS* | BPEN_589 |
| *rpe* | BVAF_572 | *rpe* | Bfl570 | *rpe* | BPEN_590 |
| *aroB* | BVAF_573 | *aroB* | Bfl571 | *aroB* | BPEN_591 |
| *aroK* | BVAF_574 | *aroK* | Bfl572 | *aroK* | BPEN_592 |
| *nfuA* | BVAF_575 | *yhgI* | Bfl573 | *gntY* | BPEN_593 |
| *asd* | BVAF_576 | *asd* | Bfl574 | *asd* | BPEN_594 |
| *yhgN* | BVAF_577 | *yhgN* | Bfl575 | *yhgN* | BPEN_595 |
| *yigL* | BVAF_578 | *yigL* | Bfl576 | *yigL* | BPEN_596 |
| *corA* | BVAF_579 | *corA* | Bfl577 | *corA* | BPEN_597 |
|  |  |  |  | *uvrD*  | BPEN_598 |
| *yigB* | BVAF_580 | *yigB* | Bfl578 | *yigB* | BPEN_599 |
| *dapF* | BVAF_581 | *dapF* | Bfl579 | *dapF* | BPEN_600 |
| *hemC* | BVAF_582 | *hemC* | Bfl580 | *hemC* | BPEN_601 |
| *hemD* | BVAF_583 | *hemD* | Bfl581 | *hemD* | BPEN_602 |
| tRNA-Pro | BVAF_584 | tRNA-Pro | Bfl582 | tRNA-Pro | BPEN_603 |
| tRNA-Leu | BVAF_585 | tRNA-Leu1 | Bfl583 | tRNA-Leu | BPEN_604 |
| tRNA-His | BVAF_586 | tRNA-His | Bfl584 | tRNA-His | BPEN_605 |
| tRNA-Arg | BVAF_587 | tRNA-Arg1 | Bfl585 | tRNA-Arg | BPEN_606 |
| *rho* | BVAF_588 | *rho* | Bfl586 | *rho* | BPEN_607 |
| *trxA* | BVAF_589 | *trxA* | Bfl587 | *trxA* | BPEN_608 |
| *ilvC* | BVAF_590 | *ilvC* | Bfl588 | *ilvC* | BPEN_609 |
| *ilvA* | BVAF_591 | *ilvA* | Bfl589 | *ilvA* | BPEN_610 |
| *ilvD* | BVAF_592 | *ilvD* | Bfl590 | *ilvD* | BPEN_611 |
| *ilvE* | BVAF_593 | *ilvE* | Bfl591 | *ilvE* | BPEN_612 |
| *ilvM* | BVAF_594 | *ilvM* | Bfl592 | *ilvM* | BPEN_613 |
| *ilvG* | BVAF_595 | *ilvG* | Bfl593 | *ilvG* | BPEN_614 |
| tRNA-Trp | BVAF_596 | tRNA-Trp | Bfl594 | tRNA-Trp | BPEN_615 |
| tRNA-Asp | BVAF_597 | tRNA-Asp | Bfl595 | tRNA-Asp | BPEN_616 |
| tRNA-Ala | BVAF_598 | tRNA-Ala | Bfl596 | tRNA-Ala | BPEN_617 |
| *murI* | BVAF_599 |  |  | *murI* | BPEN_618 |
| *metF* | BVAF_600 | *metF* | Bfl597 | *metF* | BPEN_619 |
| *metB* | BVAF_601 | *metB* | Bfl598 | *metB* | BPEN_620 |
| *rpmE* | BVAF_602 | *rpmE* | Bfl599 | *rpmE* | BPEN_621 |
| *fpr* | BVAF_603 | *fpr* | Bfl600 | *fpr* | BPEN_622 |
| *tpiA* | BVAF_604 | *tpiA* | Bfl601 | *tpiA* | BPEN_623 |
| *pfkA* | BVAF_605 | *pfkA* | Bfl602 | *pfkA* | BPEN_624 |
| *cysE* | BVAF_606 | *cysE* | Bfl603 | *cysE* | BPEN_625 |
| *gpsA* | BVAF_607 | *gpsA* | Bfl604 | *gpsA* | BPEN_626 |
|  |  |  |  | *secB* | BPEN_627 |
| *grxC* | BVAF_608 | *grxC* | Bfl605 | *grxC* | BPEN_628 |
| *yibN* | BVAF_609 | *yibN* | Bfl606 | *yibN* | BPEN_629 |
| *hldD* | BVAF_610 | *rfaD* | Bfl607 | *hldD* | BPEN_630 |
|  |  | *rfaF* | Bfl608 | *rfaF* | BPEN_631 |
| *rfaC* | BVAF_611 | *rfaC* | Bfl609 | *rfaC* | BPEN_632 |
| *kdtA* | BVAF_612 | *kdtA* | Bfl610 | *kdtA* | BPEN_633 |
|  |  |  |  | *coaD* | BPEN_634 |
| *rpmG* | BVAF_613 | *rpmG* | Bfl611 | *rpmG* | BPEN_635 |
| *rpmB* | BVAF_614 | *rpmB* | Bfl612 | *rpmB* | BPEN_636 |
|  |  |  |  | *dfp* | BPEN_637 |
| *dut* | BVAF_615 | *dut* | Bfl613 | *dut* | BPEN_638 |
| *yicC* | BVAF_616 | *yicC* | Bfl614 | *yicC* | BPEN_639 |
| BVAF_617 | BVAF_617 | unknown | Bfl615 | BPEN_640 | BPEN_640 |
| *gmk* | BVAF_618 | *gmk* | Bfl616 | *gmk* | BPEN_641 |
| *rpoZ* | BVAF_619 | *rpoZ* | Bfl617 | *rpoZ* | BPEN_642 |
|  |  | *glnA* | Bfl618 | *glnA* | BPEN_643 |
|  |  |  |  | *yihA* | BPEN_644 |
| *polA* | BVAF_620 | *polA* | Bfl619 | *polA* | BPEN_645 |
| *dsbA* | BVAF_621 |  |  | *dsbA* | BPEN_646 |
| *ubiD* | BVAF_622 | *yigC* | Bfl620 | *ubiD* | BPEN_647 |
| *ubiB* | BVAF_623 | *aarF* | Bfl621 | *ubiB* | BPEN_648 |
| *ubiE* | BVAF_624 | *ubiE* | Bfl622 | *ubiE* | BPEN_649 |
| *rmuC* | BVAF_625 | *rumC* | Bfl623 | *yigN* | BPEN_650 |
|  |  | *udp* | Bfl624 | *udp* | BPEN_651 |
| *metE* | BVAF_626 | *metE* | Bfl625 | *metE* | BPEN_652 |
| *rpoH* | BVAF_627 | *rpoH* | Bfl626 | *rpoH* | BPEN_653 |
|  |  | *ftsY* | Bfl627 | *ftsY* | BPEN_654 |
| *rsmD* | BVAF_628 | *yhhF* | Bfl628 | *yhhF* | BPEN_655 |
| *pgi* | BVAF_629 | *pgi* | Bfl629 | *pgi* | BPEN_656 |
| *metA* | BVAF_630 | *metA* | Bfl630 | *metA* | BPEN_657 |
| tRNA-Ile | BVAF_631 | tRNA-Ile | Bfl631 | tRNA-Ile | BPEN_658 |

1 *YidC* and *yidD* are fused in *B. vafer* and *B. pennsylvanicus*, but separate in *B. floridanus*.

­2 Based on comparisons with *B. vafer* and *B. pennsylvanicus*, we identified four protein-coding genes in *B. floridanus* that were not included in the original annotation.

3 Re-analysis of the *B. pennsylvanicus* genome revealed that the tRNA-Gly pseudogene reported earlier is likely functional.

4 Analysis with Rfam [1] identified *ffs*, the RNA component of the Signal Recognition Particle system, in the *B. floridanus* and *B. pennsylvanicus* genomes. This gene was not included in the original annotations of the two genomes.

1. Gardner PP, Daub J, Tate JG, Nawrocki EP, Kolbe DL, Lindgreen S, Wilkinson AC, Finn RD, Griffiths-Jones S, Eddy SR *et al*: **Rfam: updates to the RNA families database**. *Nucleic Acids Res* 2009, **37**(Database issue):D136-140.
