# Supplementary material for: Unprecedented loss of ammonia assimilation capability in a urease-encoding bacterial mutualist
Source: BMC Genomics. 2010 Dec 2;11:687. doi: 10.1186/1471-2164-11-687 (PMC3017870; doi:10.1186/1471-2164-11-687)
Supplement: Additional File 2 — Evolutionary rates analysis. Table listing the complete results of the evolutionary rates comparison. [file 1471-2164-11-687-S2.DOC]

**Additional File 2.**

Comparison of evolutionary rates in lineage leading to *B. vafer* (K_1_) and *B. floridanus* (K_2_) since their divergence from a common ancestor (denoted as "0"). *B. pennsylvanicus* was used as the outgroup. Results are sorted by K_1_/K_2_ values.

*The anomalous value for *atpE* reflects the fact that, surprisingly, *B. pennsylvanicus* and *B. vafer* show relatively low divergence for this gene, and therefore *B. pennsylvanicus* is not an appropriate outgroup for the comparison of evolutionary rates of *atpE*.

| **gene** | **dN (Bvafer-Bflor)** | **dN (Bpenn-Bflor)** | **dN (Bpenn-Bvafer)** | **K_1_ (0-Bvafer)** | **K_2_ (0-Bflor)** | **K_1_/K_2_** |
| --- | --- | --- | --- | --- | --- | --- |
| *atpE* | 0.027 | 0.051 | 0.021 | -0.003 | 0.057 | **-0.05*** |
| *csrA* | 0.046 | 0.138 | 0.100 | 0.007 | 0.084 | **0.09** |
| *prfB* | 0.111 | 0.219 | 0.134 | 0.026 | 0.196 | **0.13** |
| *rpmG* | 0.166 | 0.245 | 0.152 | 0.073 | 0.259 | **0.28** |
| *groEL* | 0.007 | 0.008 | 0.005 | 0.003 | 0.010 | **0.29** |
| *rpsJ* | 0.016 | 0.037 | 0.029 | 0.007 | 0.024 | **0.30** |
| *fabA* | 0.056 | 0.110 | 0.081 | 0.027 | 0.085 | **0.32** |
| *grxC* | 0.306 | 0.400 | 0.246 | 0.153 | 0.459 | **0.33** |
| *nusB* | 0.151 | 0.259 | 0.189 | 0.081 | 0.221 | **0.37** |
| *ispE* | 0.454 | 0.518 | 0.308 | 0.245 | 0.664 | **0.37** |
| *dxr* | 0.264 | 0.309 | 0.195 | 0.150 | 0.377 | **0.40** |
| *pal* | 0.277 | 0.408 | 0.297 | 0.166 | 0.388 | **0.43** |
| *nusG* | 0.069 | 0.089 | 0.064 | 0.044 | 0.095 | **0.46** |
| *groES* | 0.013 | 0.023 | 0.019 | 0.009 | 0.018 | **0.47** |
| *pheA* | 0.182 | 0.334 | 0.270 | 0.118 | 0.246 | **0.48** |
| *cyoD* | 0.270 | 0.359 | 0.265 | 0.176 | 0.364 | **0.48** |
| *folD* | 0.107 | 0.136 | 0.101 | 0.072 | 0.142 | **0.51** |
| *dksA* | 0.108 | 0.197 | 0.161 | 0.073 | 0.144 | **0.51** |
| *rplV* | 0.138 | 0.172 | 0.127 | 0.094 | 0.182 | **0.52** |
| *ureA* | 0.115 | 0.106 | 0.070 | 0.079 | 0.151 | **0.53** |
| *lpp* | 0.261 | 0.342 | 0.261 | 0.180 | 0.342 | **0.53** |
| *rpsR* | 0.098 | 0.142 | 0.112 | 0.068 | 0.128 | **0.54** |
| *nuoB* | 0.115 | 0.124 | 0.089 | 0.080 | 0.150 | **0.54** |
| *ybeY* | 0.195 | 0.325 | 0.267 | 0.136 | 0.253 | **0.54** |
| *ispB* | 0.114 | 0.190 | 0.157 | 0.081 | 0.147 | **0.55** |
| *cspC* | 0.024 | 0.025 | 0.018 | 0.017 | 0.030 | **0.56** |
| *mdtH* | 0.084 | 0.112 | 0.088 | 0.060 | 0.107 | **0.56** |
| *fldA* | 0.105 | 0.160 | 0.131 | 0.076 | 0.134 | **0.57** |
| *pgpA* | 0.173 | 0.290 | 0.243 | 0.126 | 0.219 | **0.58** |
| *zapA* | 0.221 | 0.228 | 0.168 | 0.162 | 0.280 | **0.58** |
| *rpsU* | 0.042 | 0.073 | 0.062 | 0.031 | 0.054 | **0.58** |
| *ahpC* | 0.112 | 0.143 | 0.114 | 0.083 | 0.142 | **0.58** |
| *birA* | 0.297 | 0.447 | 0.369 | 0.220 | 0.374 | **0.59** |
| *sufA* | 0.177 | 0.291 | 0.245 | 0.131 | 0.223 | **0.59** |
| *yigB* | 0.239 | 0.273 | 0.211 | 0.177 | 0.301 | **0.59** |
| *lplA* | 0.158 | 0.234 | 0.194 | 0.118 | 0.197 | **0.60** |
| *secY* | 0.089 | 0.114 | 0.093 | 0.068 | 0.110 | **0.62** |
| *rplT* | 0.185 | 0.225 | 0.182 | 0.141 | 0.228 | **0.62** |
| *pdxB* | 0.223 | 0.289 | 0.238 | 0.172 | 0.275 | **0.62** |
| *lpdA* | 0.197 | 0.243 | 0.197 | 0.151 | 0.243 | **0.62** |
| *ftsB* | 0.242 | 0.355 | 0.300 | 0.187 | 0.297 | **0.63** |
| *manZ* | 0.112 | 0.117 | 0.093 | 0.087 | 0.136 | **0.64** |
| *yqjA* | 0.282 | 0.331 | 0.272 | 0.223 | 0.342 | **0.65** |
| *truA* | 0.208 | 0.224 | 0.181 | 0.165 | 0.252 | **0.66** |
| *yibN* | 0.439 | 0.601 | 0.510 | 0.349 | 0.530 | **0.66** |
| *dapD* | 0.148 | 0.204 | 0.173 | 0.118 | 0.178 | **0.66** |
| *pgk* | 0.097 | 0.150 | 0.131 | 0.078 | 0.116 | **0.67** |
| *degQ* | 0.069 | 0.100 | 0.087 | 0.056 | 0.083 | **0.67** |
| *rpmJ* | 0.096 | 0.165 | 0.147 | 0.078 | 0.115 | **0.68** |
| *rplW* | 0.244 | 0.378 | 0.332 | 0.198 | 0.290 | **0.69** |
| *pgl* | 0.308 | 0.356 | 0.300 | 0.252 | 0.364 | **0.69** |
| *trpA* | 0.183 | 0.251 | 0.217 | 0.150 | 0.217 | **0.69** |
| *rpsN* | 0.143 | 0.148 | 0.122 | 0.117 | 0.168 | **0.69** |
| *murG* | 0.208 | 0.280 | 0.243 | 0.170 | 0.245 | **0.70** |
| *yeeX* | 0.137 | 0.124 | 0.099 | 0.112 | 0.161 | **0.70** |
| *rplS* | 0.131 | 0.163 | 0.140 | 0.108 | 0.154 | **0.70** |
| *rpsK* | 0.025 | 0.050 | 0.046 | 0.021 | 0.030 | **0.71** |
| *rpmF* | 0.108 | 0.175 | 0.157 | 0.090 | 0.127 | **0.71** |
| *folP* | 0.219 | 0.252 | 0.215 | 0.183 | 0.256 | **0.71** |
| *dapE* | 0.206 | 0.282 | 0.248 | 0.172 | 0.240 | **0.72** |
| *ilvG* | 0.143 | 0.149 | 0.126 | 0.119 | 0.166 | **0.72** |
| *prs* | 0.041 | 0.048 | 0.042 | 0.034 | 0.047 | **0.72** |
| *folK* | 0.287 | 0.343 | 0.297 | 0.240 | 0.333 | **0.72** |
| *lolD* | 0.201 | 0.248 | 0.215 | 0.168 | 0.233 | **0.72** |
| *rplY* | 0.512 | 0.461 | 0.379 | 0.430 | 0.595 | **0.72** |
| *sppA* | 0.154 | 0.225 | 0.200 | 0.130 | 0.179 | **0.72** |
| *omp* | 0.224 | 0.274 | 0.239 | 0.188 | 0.260 | **0.72** |
| *hemD* | 0.207 | 0.285 | 0.252 | 0.174 | 0.240 | **0.73** |
| *rpsL* | 0.060 | 0.087 | 0.077 | 0.051 | 0.070 | **0.73** |
| *ycfM* | 0.442 | 0.620 | 0.552 | 0.374 | 0.510 | **0.73** |
| *cyoA* | 0.215 | 0.246 | 0.214 | 0.182 | 0.247 | **0.74** |
| *yjgQ* | 0.288 | 0.459 | 0.416 | 0.245 | 0.331 | **0.74** |
| *proS* | 0.282 | 0.310 | 0.269 | 0.240 | 0.323 | **0.74** |
| *rpsG* | 0.084 | 0.119 | 0.106 | 0.071 | 0.096 | **0.75** |
| *plsC* | 0.208 | 0.269 | 0.238 | 0.178 | 0.238 | **0.75** |
| *rplC* | 0.210 | 0.233 | 0.202 | 0.179 | 0.240 | **0.75** |
| *nuoE* | 0.200 | 0.257 | 0.228 | 0.171 | 0.229 | **0.75** |
| *manX* | 0.159 | 0.235 | 0.213 | 0.136 | 0.182 | **0.75** |
| *nth* | 0.222 | 0.220 | 0.188 | 0.190 | 0.254 | **0.75** |
| *upp* | 0.048 | 0.075 | 0.068 | 0.041 | 0.054 | **0.75** |
| *rplJ* | 0.281 | 0.389 | 0.349 | 0.242 | 0.320 | **0.75** |
| *rpsH* | 0.169 | 0.219 | 0.195 | 0.146 | 0.193 | **0.75** |
| *tmk* | 0.233 | 0.302 | 0.270 | 0.200 | 0.265 | **0.75** |
| *clpP* | 0.142 | 0.165 | 0.145 | 0.122 | 0.161 | **0.76** |
| *holA* | 0.350 | 0.444 | 0.396 | 0.301 | 0.398 | **0.76** |
| *hisD* | 0.160 | 0.206 | 0.184 | 0.138 | 0.183 | **0.76** |
| *pnp* | 0.118 | 0.138 | 0.123 | 0.102 | 0.133 | **0.76** |
| *tolA* | 0.571 | 0.575 | 0.500 | 0.496 | 0.647 | **0.77** |
| *skp* | 0.126 | 0.196 | 0.180 | 0.109 | 0.143 | **0.77** |
| *ybeB* | 0.242 | 0.350 | 0.318 | 0.210 | 0.274 | **0.77** |
| *infB* | 0.280 | 0.336 | 0.301 | 0.244 | 0.315 | **0.77** |
| *pyrG* | 0.120 | 0.147 | 0.132 | 0.105 | 0.135 | **0.78** |
| *rplQ* | 0.208 | 0.181 | 0.155 | 0.182 | 0.234 | **0.78** |
| *rlmB* | 0.275 | 0.318 | 0.284 | 0.241 | 0.309 | **0.78** |
| *ftsQ* | 0.371 | 0.388 | 0.342 | 0.326 | 0.417 | **0.78** |
| *rpsB* | 0.076 | 0.111 | 0.102 | 0.067 | 0.085 | **0.78** |
| *mnmA* | 0.151 | 0.205 | 0.187 | 0.133 | 0.169 | **0.79** |
| *murC* | 0.212 | 0.279 | 0.255 | 0.187 | 0.236 | **0.79** |
| *mreB* | 0.021 | 0.022 | 0.020 | 0.018 | 0.023 | **0.79** |
| *rmuC* | 0.170 | 0.241 | 0.221 | 0.150 | 0.190 | **0.79** |
| *tusE* | 0.153 | 0.300 | 0.283 | 0.136 | 0.171 | **0.80** |
| *lolC* | 0.229 | 0.324 | 0.298 | 0.204 | 0.255 | **0.80** |
| *atpB* | 0.211 | 0.253 | 0.230 | 0.188 | 0.235 | **0.80** |
| *yqeI* | 0.257 | 0.310 | 0.282 | 0.229 | 0.285 | **0.80** |
| *yhgN* | 0.079 | 0.099 | 0.090 | 0.070 | 0.087 | **0.80** |
| *tonB* | 0.643 | 0.751 | 0.681 | 0.573 | 0.713 | **0.80** |
| *rplK* | 0.215 | 0.205 | 0.182 | 0.191 | 0.238 | **0.80** |
| *recD* | 0.301 | 0.376 | 0.343 | 0.269 | 0.334 | **0.81** |
| *aroE* | 0.253 | 0.301 | 0.274 | 0.226 | 0.280 | **0.81** |
| *dnaK* | 0.048 | 0.058 | 0.052 | 0.043 | 0.054 | **0.81** |
| *yjeP* | 0.265 | 0.320 | 0.292 | 0.238 | 0.293 | **0.81** |
| *cyoB* | 0.085 | 0.117 | 0.108 | 0.076 | 0.094 | **0.81** |
| *lpxB* | 0.232 | 0.269 | 0.245 | 0.208 | 0.256 | **0.81** |
| *sucC* | 0.279 | 0.332 | 0.303 | 0.251 | 0.308 | **0.81** |
| *dnaE* | 0.178 | 0.224 | 0.206 | 0.160 | 0.196 | **0.82** |
| *rpsO* | 0.186 | 0.242 | 0.223 | 0.167 | 0.205 | **0.82** |
| *eno* | 0.145 | 0.186 | 0.172 | 0.131 | 0.159 | **0.83** |
| *clpB* | 0.124 | 0.162 | 0.150 | 0.113 | 0.136 | **0.83** |
| *ligA* | 0.253 | 0.292 | 0.269 | 0.229 | 0.276 | **0.83** |
| *rluD* | 0.230 | 0.284 | 0.263 | 0.208 | 0.251 | **0.83** |
| *lnt* | 0.261 | 0.299 | 0.275 | 0.237 | 0.285 | **0.83** |
| *prfA* | 0.143 | 0.195 | 0.182 | 0.130 | 0.156 | **0.83** |
| *ubiA* | 0.289 | 0.299 | 0.273 | 0.263 | 0.316 | **0.83** |
| *dapB* | 0.260 | 0.355 | 0.331 | 0.236 | 0.284 | **0.83** |
| *amiB* | 0.300 | 0.312 | 0.286 | 0.274 | 0.326 | **0.84** |
| *ybhL* | 0.067 | 0.100 | 0.094 | 0.061 | 0.073 | **0.84** |
| *yoaE* | 0.101 | 0.133 | 0.125 | 0.093 | 0.110 | **0.84** |
| *minC* | 0.209 | 0.272 | 0.254 | 0.192 | 0.227 | **0.85** |
| *rimM* | 0.399 | 0.426 | 0.392 | 0.365 | 0.432 | **0.85** |
| *metA* | 0.117 | 0.204 | 0.195 | 0.108 | 0.127 | **0.85** |
| *ndk* | 0.172 | 0.205 | 0.191 | 0.158 | 0.186 | **0.85** |
| *yqgF* | 0.238 | 0.338 | 0.319 | 0.219 | 0.257 | **0.85** |
| *trpS* | 0.210 | 0.228 | 0.211 | 0.193 | 0.226 | **0.85** |
| *apaH* | 0.208 | 0.307 | 0.291 | 0.192 | 0.224 | **0.86** |
| *cysU* | 0.154 | 0.178 | 0.166 | 0.142 | 0.166 | **0.86** |
| *trpC* | 0.204 | 0.256 | 0.240 | 0.189 | 0.220 | **0.86** |
| *BVAF 040* | 0.259 | 0.305 | 0.285 | 0.239 | 0.278 | **0.86** |
| *nrdA* | 0.065 | 0.082 | 0.077 | 0.060 | 0.070 | **0.86** |
| *msbA* | 0.114 | 0.132 | 0.124 | 0.106 | 0.123 | **0.86** |
| *ubiB* | 0.156 | 0.178 | 0.167 | 0.145 | 0.168 | **0.86** |
| *rpmI* | 0.229 | 0.219 | 0.202 | 0.212 | 0.246 | **0.86** |
| *mraW* | 0.259 | 0.279 | 0.261 | 0.240 | 0.278 | **0.86** |
| *pheS* | 0.209 | 0.218 | 0.203 | 0.194 | 0.223 | **0.87** |
| *cysD* | 0.073 | 0.126 | 0.121 | 0.068 | 0.079 | **0.87** |
| *sdhB* | 0.109 | 0.160 | 0.152 | 0.101 | 0.117 | **0.87** |
| *zur* | 0.164 | 0.205 | 0.194 | 0.153 | 0.175 | **0.87** |
| *nuoF* | 0.115 | 0.167 | 0.159 | 0.108 | 0.123 | **0.87** |
| *clpX* | 0.177 | 0.216 | 0.204 | 0.165 | 0.189 | **0.87** |
| *tktA* | 0.156 | 0.167 | 0.156 | 0.146 | 0.167 | **0.88** |
| *holD* | 0.445 | 0.562 | 0.533 | 0.416 | 0.474 | **0.88** |
| *dapF* | 0.162 | 0.197 | 0.186 | 0.151 | 0.172 | **0.88** |
| *glmU* | 0.230 | 0.297 | 0.282 | 0.215 | 0.245 | **0.88** |
| *cysC* | 0.245 | 0.316 | 0.300 | 0.229 | 0.261 | **0.88** |
| *lptD* | 0.383 | 0.460 | 0.436 | 0.358 | 0.407 | **0.88** |
| *ptsI* | 0.116 | 0.111 | 0.104 | 0.109 | 0.123 | **0.88** |
| *lon* | 0.050 | 0.072 | 0.069 | 0.047 | 0.053 | **0.88** |
| *erfK* | 0.239 | 0.319 | 0.304 | 0.224 | 0.253 | **0.88** |
| *pgsA* | 0.237 | 0.283 | 0.269 | 0.223 | 0.251 | **0.89** |
| *aroC* | 0.104 | 0.118 | 0.112 | 0.098 | 0.110 | **0.89** |
| *hldE* | 0.197 | 0.209 | 0.197 | 0.186 | 0.209 | **0.89** |
| *purA* | 0.165 | 0.210 | 0.200 | 0.156 | 0.175 | **0.89** |
| *rpsF* | 0.292 | 0.347 | 0.330 | 0.274 | 0.309 | **0.89** |
| *grxD* | 0.183 | 0.225 | 0.215 | 0.172 | 0.194 | **0.89** |
| *nuoA* | 0.266 | 0.316 | 0.301 | 0.250 | 0.281 | **0.89** |
| *pabB* | 0.168 | 0.213 | 0.203 | 0.158 | 0.177 | **0.89** |
| *cyoE* | 0.250 | 0.289 | 0.275 | 0.236 | 0.264 | **0.89** |
| *ribD* | 0.138 | 0.177 | 0.170 | 0.130 | 0.146 | **0.90** |
| *leuB* | 0.133 | 0.163 | 0.156 | 0.126 | 0.140 | **0.90** |
| *ybeX* | 0.109 | 0.150 | 0.144 | 0.103 | 0.114 | **0.90** |
| *folA* | 0.315 | 0.368 | 0.351 | 0.299 | 0.331 | **0.90** |
| *leuC* | 0.110 | 0.143 | 0.137 | 0.104 | 0.116 | **0.90** |
| *tolB* | 0.191 | 0.229 | 0.220 | 0.181 | 0.201 | **0.90** |
| *rplF* | 0.298 | 0.332 | 0.316 | 0.282 | 0.313 | **0.90** |
| *accB* | 0.258 | 0.301 | 0.288 | 0.245 | 0.271 | **0.90** |
| *leuS* | 0.202 | 0.233 | 0.223 | 0.192 | 0.212 | **0.90** |
| *secE* | 0.329 | 0.383 | 0.367 | 0.313 | 0.345 | **0.91** |
| *nuoG* | 0.195 | 0.227 | 0.217 | 0.185 | 0.204 | **0.91** |
| *folC* | 0.230 | 0.322 | 0.311 | 0.219 | 0.241 | **0.91** |
| *sucB* | 0.224 | 0.240 | 0.229 | 0.213 | 0.235 | **0.91** |
| *pheT* | 0.271 | 0.319 | 0.305 | 0.258 | 0.284 | **0.91** |
| *nuoK* | 0.172 | 0.203 | 0.195 | 0.164 | 0.181 | **0.91** |
| *rnt* | 0.123 | 0.196 | 0.191 | 0.117 | 0.129 | **0.91** |
| *mrdA* | 0.126 | 0.149 | 0.143 | 0.120 | 0.132 | **0.91** |
| *aceE* | 0.088 | 0.119 | 0.115 | 0.085 | 0.092 | **0.92** |
| *trpE* | 0.142 | 0.175 | 0.169 | 0.136 | 0.148 | **0.92** |
| *glyA* | 0.133 | 0.175 | 0.169 | 0.127 | 0.138 | **0.92** |
| *nuoM* | 0.246 | 0.286 | 0.275 | 0.236 | 0.256 | **0.92** |
| *trpB* | 0.106 | 0.125 | 0.121 | 0.102 | 0.111 | **0.92** |
| *hisF* | 0.149 | 0.178 | 0.172 | 0.143 | 0.155 | **0.92** |
| *mnmE* | 0.208 | 0.237 | 0.229 | 0.200 | 0.216 | **0.92** |
| *dnaN* | 0.291 | 0.352 | 0.341 | 0.280 | 0.303 | **0.92** |
| *BVAF_617* | 0.056 | 0.059 | 0.057 | 0.054 | 0.058 | **0.93** |
| *none* | 0.056 | 0.059 | 0.057 | 0.054 | 0.058 | **0.93** |
| *mrdB* | 0.163 | 0.234 | 0.228 | 0.157 | 0.169 | **0.93** |
| *atpC* | 0.172 | 0.249 | 0.243 | 0.166 | 0.178 | **0.93** |
| *lgt* | 0.176 | 0.231 | 0.225 | 0.170 | 0.183 | **0.93** |
| *obgE* | 0.299 | 0.289 | 0.278 | 0.288 | 0.309 | **0.93** |
| *mutY* | 0.302 | 0.366 | 0.355 | 0.292 | 0.313 | **0.93** |
| *tilS* | 0.286 | 0.340 | 0.330 | 0.276 | 0.296 | **0.93** |
| *fbaA* | 0.133 | 0.135 | 0.130 | 0.128 | 0.137 | **0.94** |
| *rpoD* | 0.075 | 0.092 | 0.089 | 0.073 | 0.078 | **0.94** |
| *fabG* | 0.161 | 0.164 | 0.159 | 0.156 | 0.166 | **0.94** |
| *tgt* | 0.170 | 0.189 | 0.183 | 0.165 | 0.175 | **0.94** |
| *rpsI* | 0.166 | 0.183 | 0.178 | 0.161 | 0.171 | **0.94** |
| *gmhA* | 0.205 | 0.213 | 0.207 | 0.199 | 0.211 | **0.94** |
| *ybeZ* | 0.157 | 0.214 | 0.210 | 0.152 | 0.161 | **0.94** |
| *BVAF 018* | 0.198 | 0.208 | 0.202 | 0.192 | 0.204 | **0.94** |
| *pdxA* | 0.180 | 0.229 | 0.224 | 0.175 | 0.185 | **0.94** |
| *ribH* | 0.218 | 0.220 | 0.214 | 0.212 | 0.224 | **0.95** |
| *hisI* | 0.120 | 0.183 | 0.180 | 0.117 | 0.123 | **0.95** |
| *ygiH* | 0.274 | 0.320 | 0.313 | 0.267 | 0.282 | **0.95** |
| *yeaZ* | 0.278 | 0.354 | 0.347 | 0.271 | 0.285 | **0.95** |
| *metF* | 0.147 | 0.147 | 0.144 | 0.144 | 0.151 | **0.95** |
| *metE* | 0.174 | 0.210 | 0.206 | 0.170 | 0.178 | **0.95** |
| *trpD* | 0.261 | 0.325 | 0.319 | 0.255 | 0.267 | **0.96** |
| *rplD* | 0.242 | 0.243 | 0.238 | 0.236 | 0.247 | **0.96** |
| *ureG* | 0.172 | 0.149 | 0.146 | 0.168 | 0.176 | **0.96** |
| *tsf* | 0.251 | 0.283 | 0.277 | 0.245 | 0.256 | **0.96** |
| *trxB* | 0.176 | 0.166 | 0.162 | 0.173 | 0.180 | **0.96** |
| *pykA* | 0.128 | 0.150 | 0.147 | 0.126 | 0.131 | **0.96** |
| *leuA* | 0.150 | 0.147 | 0.144 | 0.147 | 0.153 | **0.96** |
| *ytfN* | 0.501 | 0.527 | 0.518 | 0.492 | 0.510 | **0.97** |
| *rnhB* | 0.233 | 0.259 | 0.255 | 0.230 | 0.237 | **0.97** |
| *sufE* | 0.289 | 0.333 | 0.328 | 0.284 | 0.293 | **0.97** |
| *alaS* | 0.213 | 0.253 | 0.250 | 0.210 | 0.216 | **0.97** |
| *BVAF_037* | 0.684 | 0.734 | 0.724 | 0.674 | 0.695 | **0.97** |
| *atpG* | 0.159 | 0.205 | 0.203 | 0.157 | 0.161 | **0.97** |
| *cysJ* | 0.258 | 0.258 | 0.255 | 0.255 | 0.262 | **0.97** |
| *cysP* | 0.160 | 0.190 | 0.188 | 0.158 | 0.162 | **0.98** |
| *frr* | 0.254 | 0.263 | 0.260 | 0.251 | 0.257 | **0.98** |
| *yqgE* | 0.104 | 0.142 | 0.141 | 0.103 | 0.105 | **0.98** |
| *engD* | 0.281 | 0.306 | 0.304 | 0.279 | 0.284 | **0.98** |
| *ilvD* | 0.118 | 0.121 | 0.120 | 0.117 | 0.119 | **0.98** |
| *rnc* | 0.231 | 0.243 | 0.241 | 0.229 | 0.233 | **0.98** |
| *pssA* | 0.176 | 0.254 | 0.252 | 0.174 | 0.177 | **0.98** |
| *mraY* | 0.320 | 0.345 | 0.342 | 0.317 | 0.322 | **0.98** |
| *mnmG* | 0.120 | 0.153 | 0.152 | 0.119 | 0.121 | **0.98** |
| *secD* | 0.202 | 0.286 | 0.285 | 0.201 | 0.204 | **0.99** |
| *aroQ* | 0.162 | 0.157 | 0.156 | 0.161 | 0.163 | **0.99** |
| *rplE* | 0.119 | 0.141 | 0.140 | 0.118 | 0.119 | **0.99** |
| *pyrH* | 0.121 | 0.141 | 0.140 | 0.120 | 0.122 | **0.99** |
| *purH* | 0.250 | 0.262 | 0.260 | 0.248 | 0.251 | **0.99** |
| *ubiG* | 0.210 | 0.283 | 0.282 | 0.209 | 0.211 | **0.99** |
| *atpD* | 0.079 | 0.090 | 0.089 | 0.079 | 0.079 | **0.99** |
| *minE* | 0.331 | 0.404 | 0.403 | 0.330 | 0.333 | **0.99** |
| *ubiH* | 0.301 | 0.331 | 0.330 | 0.300 | 0.302 | **0.99** |
| *dxs* | 0.107 | 0.145 | 0.145 | 0.106 | 0.107 | **0.99** |
| *cutA* | 0.383 | 0.426 | 0.425 | 0.383 | 0.384 | **1.00** |
| *nuoN* | 0.290 | 0.367 | 0.366 | 0.290 | 0.290 | **1.00** |
| *thrB* | 0.166 | 0.214 | 0.214 | 0.166 | 0.166 | **1.00** |
| *ptsH* | 0.108 | 0.163 | 0.163 | 0.108 | 0.108 | **1.00** |
| *deaD* | 0.241 | 0.271 | 0.271 | 0.241 | 0.241 | **1.00** |
| *mreD* | 0.360 | 0.370 | 0.371 | 0.360 | 0.359 | **1.00** |
| *thiI* | 0.223 | 0.283 | 0.283 | 0.223 | 0.223 | **1.00** |
| *hemC* | 0.131 | 0.155 | 0.155 | 0.131 | 0.131 | **1.01** |
| *rplA* | 0.258 | 0.227 | 0.228 | 0.259 | 0.257 | **1.01** |
| *thrS* | 0.135 | 0.178 | 0.179 | 0.136 | 0.135 | **1.01** |
| *murE* | 0.235 | 0.284 | 0.285 | 0.236 | 0.234 | **1.01** |
| *cmk* | 0.234 | 0.245 | 0.246 | 0.235 | 0.233 | **1.01** |
| *lysA* | 0.150 | 0.166 | 0.167 | 0.151 | 0.149 | **1.01** |
| *dnaB* | 0.117 | 0.140 | 0.141 | 0.118 | 0.117 | **1.01** |
| *ileS* | 0.203 | 0.232 | 0.233 | 0.204 | 0.202 | **1.01** |
| *fabD* | 0.231 | 0.281 | 0.283 | 0.233 | 0.229 | **1.02** |
| *leuD* | 0.225 | 0.224 | 0.226 | 0.226 | 0.223 | **1.02** |
| *yraL* | 0.191 | 0.226 | 0.228 | 0.193 | 0.190 | **1.02** |
| *gyrB* | 0.092 | 0.117 | 0.118 | 0.093 | 0.091 | **1.02** |
| *pgi* | 0.139 | 0.166 | 0.168 | 0.141 | 0.137 | **1.03** |
| *polA* | 0.186 | 0.213 | 0.215 | 0.188 | 0.183 | **1.03** |
| *lpxH* | 0.223 | 0.284 | 0.287 | 0.226 | 0.220 | **1.03** |
| *surA* | 0.369 | 0.497 | 0.502 | 0.374 | 0.365 | **1.03** |
| *fabH* | 0.107 | 0.120 | 0.121 | 0.108 | 0.106 | **1.03** |
| *sufS* | 0.159 | 0.175 | 0.177 | 0.161 | 0.157 | **1.03** |
| *gltP* | 0.057 | 0.059 | 0.060 | 0.058 | 0.056 | **1.03** |
| *trpG* | 0.143 | 0.192 | 0.194 | 0.145 | 0.141 | **1.03** |
| *dnaG* | 0.193 | 0.255 | 0.258 | 0.196 | 0.190 | **1.03** |
| *ytfM* | 0.266 | 0.331 | 0.335 | 0.270 | 0.261 | **1.03** |
| *guaA* | 0.224 | 0.249 | 0.253 | 0.227 | 0.220 | **1.03** |
| *ung* | 0.164 | 0.205 | 0.208 | 0.167 | 0.161 | **1.03** |
| *yhcB* | 0.344 | 0.358 | 0.364 | 0.350 | 0.338 | **1.04** |
| *yfjG* | 0.266 | 0.358 | 0.363 | 0.271 | 0.261 | **1.04** |
| *tyrA* | 0.193 | 0.214 | 0.218 | 0.197 | 0.190 | **1.04** |
| *nagA* | 0.163 | 0.212 | 0.215 | 0.166 | 0.159 | **1.04** |
| *prlC* | 0.320 | 0.357 | 0.363 | 0.326 | 0.314 | **1.04** |
| *nagB* | 0.146 | 0.179 | 0.182 | 0.149 | 0.143 | **1.04** |
| *serS* | 0.174 | 0.226 | 0.230 | 0.178 | 0.170 | **1.04** |
| *dsbB* | 0.373 | 0.452 | 0.460 | 0.381 | 0.365 | **1.04** |
| *sucA* | 0.215 | 0.250 | 0.255 | 0.220 | 0.211 | **1.04** |
| *hflK* | 0.224 | 0.224 | 0.229 | 0.229 | 0.219 | **1.05** |
| *kdsA* | 0.167 | 0.185 | 0.188 | 0.170 | 0.163 | **1.05** |
| *lysS* | 0.203 | 0.213 | 0.218 | 0.208 | 0.198 | **1.05** |
| *argI* | 0.137 | 0.143 | 0.146 | 0.141 | 0.134 | **1.05** |
| *hisB* | 0.149 | 0.161 | 0.164 | 0.152 | 0.145 | **1.05** |
| *ygfZ* | 0.298 | 0.377 | 0.385 | 0.306 | 0.291 | **1.05** |
| *ribE* | 0.194 | 0.221 | 0.226 | 0.199 | 0.189 | **1.05** |
| *sucD* | 0.123 | 0.150 | 0.153 | 0.126 | 0.119 | **1.05** |
| *rpsA* | 0.158 | 0.158 | 0.162 | 0.162 | 0.154 | **1.06** |
| *ycbL* | 0.197 | 0.250 | 0.256 | 0.203 | 0.191 | **1.06** |
| *aroK* | 0.192 | 0.164 | 0.170 | 0.198 | 0.186 | **1.07** |
| *hinT* | 0.134 | 0.137 | 0.141 | 0.139 | 0.130 | **1.07** |
| *yjcE* | 0.058 | 0.077 | 0.079 | 0.060 | 0.056 | **1.07** |
| *nuoI* | 0.128 | 0.143 | 0.147 | 0.133 | 0.124 | **1.07** |
| *sodA* | 0.239 | 0.238 | 0.247 | 0.248 | 0.231 | **1.07** |
| *hldD* | 0.218 | 0.246 | 0.254 | 0.226 | 0.210 | **1.08** |
| *accC* | 0.119 | 0.142 | 0.147 | 0.123 | 0.114 | **1.08** |
| *pabA* | 0.134 | 0.178 | 0.183 | 0.139 | 0.129 | **1.08** |
| *tyrS* | 0.187 | 0.231 | 0.238 | 0.194 | 0.180 | **1.08** |
| *glyS* | 0.296 | 0.390 | 0.402 | 0.307 | 0.285 | **1.08** |
| *murB* | 0.212 | 0.252 | 0.260 | 0.220 | 0.203 | **1.08** |
| *pabC* | 0.257 | 0.260 | 0.270 | 0.267 | 0.246 | **1.08** |
| *adk* | 0.256 | 0.322 | 0.332 | 0.267 | 0.246 | **1.09** |
| *aspS* | 0.192 | 0.206 | 0.214 | 0.200 | 0.184 | **1.09** |
| *ftsH* | 0.062 | 0.083 | 0.086 | 0.065 | 0.060 | **1.09** |
| *ftsI* | 0.189 | 0.206 | 0.214 | 0.197 | 0.181 | **1.09** |
| *yjeE* | 0.299 | 0.365 | 0.377 | 0.312 | 0.287 | **1.09** |
| *rpoB* | 0.080 | 0.084 | 0.088 | 0.083 | 0.076 | **1.09** |
| *suhB* | 0.332 | 0.399 | 0.414 | 0.347 | 0.317 | **1.09** |
| *mreC* | 0.262 | 0.273 | 0.285 | 0.274 | 0.250 | **1.09** |
| *kdsB* | 0.245 | 0.296 | 0.307 | 0.256 | 0.233 | **1.10** |
| *cysH* | 0.212 | 0.224 | 0.234 | 0.221 | 0.202 | **1.10** |
| *rlmE* | 0.279 | 0.302 | 0.316 | 0.292 | 0.266 | **1.10** |
| *cysI* | 0.117 | 0.149 | 0.155 | 0.123 | 0.111 | **1.11** |
| *recC* | 0.296 | 0.392 | 0.407 | 0.311 | 0.281 | **1.11** |
| *folE* | 0.078 | 0.118 | 0.122 | 0.082 | 0.074 | **1.11** |
| *gcp* | 0.167 | 0.190 | 0.199 | 0.176 | 0.158 | **1.11** |
| *argS* | 0.194 | 0.222 | 0.232 | 0.204 | 0.184 | **1.11** |
| *tldD* | 0.165 | 0.179 | 0.188 | 0.174 | 0.157 | **1.11** |
| *rsmD* | 0.335 | 0.334 | 0.351 | 0.353 | 0.317 | **1.11** |
| *yfcB* | 0.207 | 0.236 | 0.247 | 0.218 | 0.196 | **1.11** |
| *rpsQ* | 0.223 | 0.233 | 0.246 | 0.235 | 0.210 | **1.12** |
| *cysN* | 0.182 | 0.235 | 0.245 | 0.192 | 0.172 | **1.12** |
| *pmbA* | 0.214 | 0.224 | 0.236 | 0.226 | 0.202 | **1.12** |
| *rpoC* | 0.059 | 0.073 | 0.076 | 0.062 | 0.056 | **1.12** |
| *metB* | 0.178 | 0.158 | 0.169 | 0.189 | 0.168 | **1.12** |
| *yajR* | 0.270 | 0.378 | 0.394 | 0.285 | 0.254 | **1.12** |
| *ureD* | 0.336 | 0.317 | 0.337 | 0.356 | 0.316 | **1.13** |
| *engA* | 0.364 | 0.378 | 0.400 | 0.386 | 0.342 | **1.13** |
| *iscS* | 0.122 | 0.129 | 0.137 | 0.129 | 0.114 | **1.13** |
| *yggX* | 0.281 | 0.258 | 0.275 | 0.298 | 0.263 | **1.13** |
| *yebA* | 0.274 | 0.316 | 0.333 | 0.291 | 0.257 | **1.13** |
| *carB* | 0.126 | 0.155 | 0.163 | 0.134 | 0.118 | **1.14** |
| *BVAF 041* | 0.170 | 0.173 | 0.184 | 0.181 | 0.159 | **1.14** |
| *nusA* | 0.120 | 0.155 | 0.163 | 0.127 | 0.112 | **1.14** |
| *ytfF* | 0.184 | 0.207 | 0.219 | 0.197 | 0.172 | **1.14** |
| *valS* | 0.246 | 0.288 | 0.304 | 0.262 | 0.229 | **1.14** |
| *zwf* | 0.142 | 0.186 | 0.195 | 0.152 | 0.133 | **1.15** |
| *fabI* | 0.175 | 0.170 | 0.182 | 0.187 | 0.163 | **1.15** |
| *ilvE* | 0.166 | 0.165 | 0.176 | 0.178 | 0.155 | **1.15** |
| *glmM* | 0.110 | 0.121 | 0.129 | 0.118 | 0.103 | **1.15** |
| *yjgF* | 0.306 | 0.331 | 0.352 | 0.327 | 0.284 | **1.15** |
| *pit* | 0.145 | 0.173 | 0.184 | 0.155 | 0.135 | **1.15** |
| *gyrA* | 0.094 | 0.108 | 0.114 | 0.100 | 0.087 | **1.15** |
| *yidZ* | 0.124 | 0.156 | 0.165 | 0.133 | 0.114 | **1.16** |
| *cls* | 0.142 | 0.156 | 0.166 | 0.152 | 0.131 | **1.16** |
| *hisS* | 0.186 | 0.204 | 0.217 | 0.200 | 0.172 | **1.16** |
| *rho* | 0.011 | 0.011 | 0.012 | 0.011 | 0.010 | **1.16** |
| *folB* | 0.482 | 0.392 | 0.429 | 0.519 | 0.446 | **1.16** |
| *sbcB* | 0.265 | 0.301 | 0.321 | 0.285 | 0.245 | **1.16** |
| *cysS* | 0.204 | 0.225 | 0.241 | 0.220 | 0.188 | **1.17** |
| *truB* | 0.213 | 0.256 | 0.273 | 0.230 | 0.195 | **1.18** |
| *thrA* | 0.140 | 0.148 | 0.159 | 0.151 | 0.128 | **1.18** |
| *cysW* | 0.190 | 0.229 | 0.245 | 0.205 | 0.174 | **1.18** |
| *tdk* | 0.157 | 0.171 | 0.184 | 0.170 | 0.144 | **1.18** |
| *cysE* | 0.164 | 0.180 | 0.194 | 0.178 | 0.151 | **1.18** |
| *nuoL* | 0.296 | 0.279 | 0.304 | 0.321 | 0.272 | **1.18** |
| *mviN* | 0.181 | 0.246 | 0.261 | 0.196 | 0.166 | **1.18** |
| *lepB* | 0.180 | 0.258 | 0.273 | 0.195 | 0.165 | **1.18** |
| *hisH* | 0.208 | 0.210 | 0.228 | 0.226 | 0.191 | **1.18** |
| *asd* | 0.158 | 0.180 | 0.193 | 0.172 | 0.145 | **1.18** |
| *atpF* | 0.347 | 0.310 | 0.339 | 0.376 | 0.318 | **1.18** |
| *pdxJ* | 0.207 | 0.199 | 0.216 | 0.224 | 0.190 | **1.18** |
| *kdtA* | 0.304 | 0.303 | 0.329 | 0.330 | 0.278 | **1.19** |
| *ureF* | 0.219 | 0.259 | 0.278 | 0.237 | 0.200 | **1.19** |
| *dut* | 0.237 | 0.198 | 0.218 | 0.258 | 0.217 | **1.19** |
| *gmk* | 0.235 | 0.223 | 0.244 | 0.255 | 0.214 | **1.19** |
| *cysA* | 0.113 | 0.122 | 0.132 | 0.123 | 0.103 | **1.19** |
| *thrC* | 0.171 | 0.176 | 0.191 | 0.186 | 0.156 | **1.19** |
| *tolQ* | 0.218 | 0.235 | 0.254 | 0.238 | 0.199 | **1.19** |
| *gpmA* | 0.188 | 0.205 | 0.221 | 0.204 | 0.171 | **1.20** |
| *nuoH* | 0.155 | 0.181 | 0.195 | 0.169 | 0.141 | **1.20** |
| *metK* | 0.127 | 0.140 | 0.152 | 0.138 | 0.115 | **1.20** |
| *infA* | 0.030 | 0.055 | 0.058 | 0.033 | 0.027 | **1.20** |
| *rplN* | 0.086 | 0.070 | 0.078 | 0.094 | 0.079 | **1.20** |
| *lepA* | 0.190 | 0.191 | 0.208 | 0.207 | 0.172 | **1.20** |
| *aroA* | 0.163 | 0.181 | 0.196 | 0.178 | 0.148 | **1.21** |
| *cysK* | 0.117 | 0.124 | 0.134 | 0.127 | 0.106 | **1.21** |
| *minD* | 0.110 | 0.102 | 0.112 | 0.120 | 0.099 | **1.21** |
| *uppS* | 0.156 | 0.139 | 0.154 | 0.170 | 0.141 | **1.21** |
| *gpsA* | 0.198 | 0.230 | 0.249 | 0.216 | 0.179 | **1.21** |
| *rplB* | 0.122 | 0.114 | 0.126 | 0.134 | 0.111 | **1.21** |
| *cysG* | 0.176 | 0.212 | 0.229 | 0.192 | 0.159 | **1.21** |
| *yrdC* | 0.206 | 0.199 | 0.219 | 0.225 | 0.186 | **1.21** |
| *hemK* | 0.262 | 0.262 | 0.287 | 0.287 | 0.237 | **1.21** |
| *yrbA* | 0.365 | 0.389 | 0.424 | 0.400 | 0.330 | **1.21** |
| *ubiF* | 0.309 | 0.276 | 0.306 | 0.339 | 0.279 | **1.21** |
| *gnd* | 0.191 | 0.204 | 0.222 | 0.210 | 0.173 | **1.21** |
| *acpS* | 0.193 | 0.202 | 0.221 | 0.212 | 0.174 | **1.22** |
| *rpsD* | 0.092 | 0.119 | 0.128 | 0.101 | 0.083 | **1.22** |
| *rpoA* | 0.093 | 0.112 | 0.121 | 0.102 | 0.083 | **1.22** |
| *ppa* | 0.193 | 0.181 | 0.201 | 0.212 | 0.173 | **1.22** |
| *sdhC* | 0.250 | 0.263 | 0.289 | 0.275 | 0.225 | **1.22** |
| *nuoJ* | 0.306 | 0.390 | 0.421 | 0.337 | 0.275 | **1.23** |
| *rnhA* | 0.092 | 0.099 | 0.109 | 0.101 | 0.082 | **1.23** |
| *map* | 0.134 | 0.145 | 0.158 | 0.148 | 0.120 | **1.23** |
| *ubiX* | 0.160 | 0.163 | 0.179 | 0.176 | 0.144 | **1.23** |
| *ureC* | 0.103 | 0.110 | 0.120 | 0.113 | 0.092 | **1.23** |
| *oprC* | 0.382 | 0.383 | 0.423 | 0.422 | 0.343 | **1.23** |
| *lspA* | 0.419 | 0.380 | 0.424 | 0.463 | 0.376 | **1.23** |
| *dnaJ* | 0.073 | 0.081 | 0.089 | 0.081 | 0.066 | **1.23** |
| *plsX* | 0.141 | 0.159 | 0.174 | 0.156 | 0.126 | **1.23** |
| *rseP* | 0.327 | 0.371 | 0.405 | 0.361 | 0.293 | **1.23** |
| *lpxK* | 0.176 | 0.178 | 0.197 | 0.194 | 0.157 | **1.24** |
| *rne* | 0.151 | 0.177 | 0.194 | 0.167 | 0.135 | **1.24** |
| *rpe* | 0.183 | 0.181 | 0.200 | 0.203 | 0.164 | **1.24** |
| *accD* | 0.108 | 0.145 | 0.157 | 0.120 | 0.096 | **1.24** |
| *atpA* | 0.112 | 0.114 | 0.126 | 0.124 | 0.100 | **1.24** |
| *rplM* | 0.187 | 0.264 | 0.285 | 0.207 | 0.166 | **1.25** |
| *murA* | 0.095 | 0.115 | 0.126 | 0.105 | 0.084 | **1.25** |
| *metG* | 0.146 | 0.182 | 0.199 | 0.163 | 0.130 | **1.26** |
| *nuoC* | 0.097 | 0.126 | 0.137 | 0.108 | 0.086 | **1.26** |
| *aroB* | 0.222 | 0.214 | 0.239 | 0.247 | 0.197 | **1.26** |
| *fmt* | 0.251 | 0.271 | 0.300 | 0.280 | 0.221 | **1.27** |
| *smpB* | 0.268 | 0.309 | 0.340 | 0.299 | 0.236 | **1.27** |
| *yaeT* | 0.299 | 0.301 | 0.337 | 0.334 | 0.263 | **1.27** |
| *holC* | 0.334 | 0.370 | 0.409 | 0.373 | 0.294 | **1.27** |
| *emtA* | 0.284 | 0.258 | 0.291 | 0.317 | 0.250 | **1.27** |
| *hisA* | 0.170 | 0.215 | 0.236 | 0.190 | 0.149 | **1.27** |
| *gmhB* | 0.232 | 0.266 | 0.294 | 0.260 | 0.204 | **1.27** |
| *glnS* | 0.165 | 0.159 | 0.178 | 0.185 | 0.145 | **1.27** |
| *cysQ* | 0.196 | 0.191 | 0.215 | 0.220 | 0.173 | **1.27** |
| *metC* | 0.156 | 0.157 | 0.175 | 0.174 | 0.137 | **1.27** |
| *ksgA* | 0.206 | 0.246 | 0.272 | 0.232 | 0.180 | **1.28** |
| *nrdB* | 0.060 | 0.063 | 0.070 | 0.068 | 0.053 | **1.29** |
| *serC* | 0.205 | 0.227 | 0.253 | 0.231 | 0.179 | **1.29** |
| *fumC* | 0.141 | 0.172 | 0.189 | 0.159 | 0.123 | **1.29** |
| *greA* | 0.167 | 0.149 | 0.170 | 0.189 | 0.146 | **1.29** |
| *lolE* | 0.258 | 0.339 | 0.372 | 0.291 | 0.225 | **1.29** |
| *ftsZ* | 0.081 | 0.057 | 0.067 | 0.091 | 0.071 | **1.29** |
| *nlpD* | 0.332 | 0.399 | 0.442 | 0.375 | 0.290 | **1.29** |
| *purB* | 0.122 | 0.152 | 0.168 | 0.138 | 0.107 | **1.29** |
| *rplI* | 0.309 | 0.278 | 0.318 | 0.349 | 0.269 | **1.29** |
| *ibpA* | 0.068 | 0.081 | 0.090 | 0.076 | 0.059 | **1.30** |
| *ribB* | 0.109 | 0.117 | 0.131 | 0.123 | 0.095 | **1.30** |
| *yicC* | 0.374 | 0.339 | 0.388 | 0.423 | 0.325 | **1.30** |
| *recB* | 0.306 | 0.339 | 0.380 | 0.347 | 0.266 | **1.30** |
| *ftsA* | 0.074 | 0.071 | 0.081 | 0.083 | 0.064 | **1.30** |
| *ftsK* | 0.362 | 0.421 | 0.470 | 0.410 | 0.314 | **1.31** |
| *dnaX* | 0.312 | 0.322 | 0.364 | 0.354 | 0.270 | **1.31** |
| *tal* | 0.440 | 1.046 | 1.105 | 0.500 | 0.381 | **1.31** |
| *rpoH* | 0.070 | 0.109 | 0.119 | 0.079 | 0.060 | **1.32** |
| *xthA* | 0.212 | 0.196 | 0.225 | 0.241 | 0.183 | **1.32** |
| *sdhD* | 0.348 | 0.430 | 0.478 | 0.396 | 0.300 | **1.32** |
| *aceF* | 0.244 | 0.241 | 0.275 | 0.278 | 0.211 | **1.32** |
| *ddl* | 0.267 | 0.313 | 0.351 | 0.304 | 0.230 | **1.32** |
| *accA* | 0.124 | 0.120 | 0.137 | 0.142 | 0.107 | **1.33** |
| *hflX* | 0.179 | 0.285 | 0.310 | 0.204 | 0.153 | **1.33** |
| *cdsA* | 0.277 | 0.370 | 0.409 | 0.316 | 0.238 | **1.33** |
| *yjgP* | 0.431 | 0.477 | 0.539 | 0.493 | 0.369 | **1.34** |
| *tuf* | 0.038 | 0.057 | 0.062 | 0.043 | 0.032 | **1.35** |
| *def* | 0.160 | 0.194 | 0.217 | 0.183 | 0.136 | **1.35** |
| *miaA* | 0.226 | 0.261 | 0.295 | 0.259 | 0.192 | **1.35** |
| *asnS* | 0.173 | 0.193 | 0.218 | 0.199 | 0.147 | **1.35** |
| *tadA* | 0.294 | 0.292 | 0.336 | 0.337 | 0.250 | **1.35** |
| *pepA* | 0.204 | 0.246 | 0.276 | 0.235 | 0.173 | **1.36** |
| *rpmB* | 0.166 | 0.190 | 0.215 | 0.191 | 0.141 | **1.36** |
| *efp* | 0.218 | 0.276 | 0.309 | 0.251 | 0.185 | **1.36** |
| *rbfA* | 0.182 | 0.289 | 0.317 | 0.209 | 0.154 | **1.36** |
| *sufC* | 0.088 | 0.123 | 0.137 | 0.102 | 0.075 | **1.36** |
| *secA* | 0.131 | 0.120 | 0.140 | 0.152 | 0.111 | **1.37** |
| *yhbG* | 0.171 | 0.178 | 0.204 | 0.198 | 0.144 | **1.37** |
| *yajC* | 0.131 | 0.218 | 0.239 | 0.152 | 0.110 | **1.37** |
| *dapA* | 0.122 | 0.130 | 0.149 | 0.142 | 0.103 | **1.38** |
| *murF* | 0.242 | 0.255 | 0.294 | 0.282 | 0.203 | **1.39** |
| *carA* | 0.172 | 0.186 | 0.214 | 0.200 | 0.144 | **1.39** |
| *mrcB* | 0.169 | 0.217 | 0.244 | 0.196 | 0.141 | **1.39** |
| *aroF* | 0.181 | 0.191 | 0.220 | 0.211 | 0.151 | **1.39** |
| *erpA* | 0.270 | 0.225 | 0.269 | 0.315 | 0.226 | **1.39** |
| *sdhA* | 0.086 | 0.085 | 0.099 | 0.100 | 0.071 | **1.40** |
| *ygfA* | 0.252 | 0.308 | 0.350 | 0.295 | 0.210 | **1.41** |
| *cyoC* | 0.240 | 0.307 | 0.348 | 0.280 | 0.199 | **1.41** |
| *ssb* | 0.218 | 0.199 | 0.237 | 0.255 | 0.180 | **1.41** |
| *ubiD* | 0.125 | 0.125 | 0.147 | 0.146 | 0.103 | **1.42** |
| *ftsW* | 0.170 | 0.181 | 0.210 | 0.200 | 0.141 | **1.42** |
| *pfkA* | 0.101 | 0.095 | 0.113 | 0.119 | 0.084 | **1.42** |
| *aspC* | 0.174 | 0.216 | 0.246 | 0.205 | 0.144 | **1.42** |
| *ubiE* | 0.185 | 0.168 | 0.200 | 0.218 | 0.153 | **1.42** |
| *grpE* | 0.136 | 0.171 | 0.195 | 0.160 | 0.112 | **1.43** |
| *guaB* | 0.096 | 0.115 | 0.132 | 0.113 | 0.079 | **1.43** |
| *ribA* | 0.126 | 0.109 | 0.131 | 0.148 | 0.103 | **1.43** |
| *rfaC* | 0.258 | 0.269 | 0.315 | 0.304 | 0.212 | **1.43** |
| *mqo* | 0.215 | 0.247 | 0.285 | 0.253 | 0.177 | **1.44** |
| *ilvM* | 0.247 | 0.210 | 0.254 | 0.291 | 0.203 | **1.44** |
| *rpiA* | 0.129 | 0.132 | 0.155 | 0.152 | 0.106 | **1.44** |
| *cca* | 0.192 | 0.242 | 0.277 | 0.227 | 0.158 | **1.44** |
| *ilvA* | 0.104 | 0.095 | 0.114 | 0.122 | 0.085 | **1.44** |
| *rpsM* | 0.106 | 0.110 | 0.129 | 0.125 | 0.087 | **1.45** |
| *pdxH* | 0.170 | 0.196 | 0.228 | 0.202 | 0.138 | **1.46** |
| *gap* | 0.111 | 0.138 | 0.159 | 0.133 | 0.090 | **1.47** |
| *mntH* | 0.076 | 0.095 | 0.110 | 0.090 | 0.061 | **1.48** |
| *lolA* | 0.401 | 0.395 | 0.473 | 0.479 | 0.323 | **1.48** |
| *yraP* | 0.208 | 0.280 | 0.321 | 0.248 | 0.167 | **1.49** |
| *infC* | 0.111 | 0.108 | 0.130 | 0.133 | 0.089 | **1.50** |
| *orn* | 0.168 | 0.182 | 0.216 | 0.202 | 0.134 | **1.50** |
| *trmD* | 0.203 | 0.190 | 0.231 | 0.243 | 0.162 | **1.50** |
| *fpr* | 0.215 | 0.251 | 0.294 | 0.259 | 0.172 | **1.50** |
| *rplR* | 0.201 | 0.191 | 0.232 | 0.242 | 0.160 | **1.51** |
| *pth* | 0.230 | 0.330 | 0.378 | 0.278 | 0.183 | **1.52** |
| *ppnK* | 0.182 | 0.157 | 0.196 | 0.221 | 0.144 | **1.54** |
| *manY* | 0.080 | 0.120 | 0.137 | 0.097 | 0.063 | **1.55** |
| *gltX* | 0.256 | 0.243 | 0.298 | 0.311 | 0.201 | **1.55** |
| *nfuA* | 0.242 | 0.344 | 0.396 | 0.294 | 0.190 | **1.55** |
| *rplP* | 0.076 | 0.099 | 0.116 | 0.093 | 0.060 | **1.55** |
| *rplU* | 0.246 | 0.253 | 0.307 | 0.301 | 0.192 | **1.57** |
| *holB* | 0.329 | 0.328 | 0.401 | 0.403 | 0.256 | **1.58** |
| *rpmH* | 0.112 | 0.144 | 0.169 | 0.137 | 0.086 | **1.58** |
| *ftsL* | 0.275 | 0.263 | 0.327 | 0.340 | 0.211 | **1.61** |
| *hflC* | 0.102 | 0.118 | 0.142 | 0.126 | 0.078 | **1.61** |
| *yfiO* | 0.213 | 0.301 | 0.351 | 0.263 | 0.163 | **1.62** |
| *smpA* | 0.433 | 0.665 | 0.768 | 0.535 | 0.331 | **1.62** |
| *fabB* | 0.117 | 0.096 | 0.124 | 0.145 | 0.090 | **1.62** |
| *ilvC* | 0.134 | 0.144 | 0.176 | 0.165 | 0.102 | **1.62** |
| *sufB* | 0.092 | 0.104 | 0.126 | 0.114 | 0.070 | **1.63** |
| *gutQ* | 0.170 | 0.134 | 0.175 | 0.211 | 0.129 | **1.63** |
| *secF* | 0.303 | 0.340 | 0.414 | 0.377 | 0.230 | **1.64** |
| *fusA* | 0.081 | 0.090 | 0.110 | 0.101 | 0.061 | **1.65** |
| *psd* | 0.178 | 0.189 | 0.233 | 0.222 | 0.134 | **1.65** |
| *sufD* | 0.320 | 0.314 | 0.393 | 0.400 | 0.240 | **1.66** |
| *yfaE* | 0.307 | 0.262 | 0.338 | 0.384 | 0.230 | **1.66** |
| *corA* | 0.166 | 0.207 | 0.248 | 0.207 | 0.124 | **1.67** |
| *lpxC* | 0.200 | 0.185 | 0.235 | 0.251 | 0.150 | **1.67** |
| *trxA* | 0.213 | 0.236 | 0.290 | 0.266 | 0.159 | **1.67** |
| *rplX* | 0.197 | 0.157 | 0.207 | 0.247 | 0.148 | **1.68** |
| *ycfH* | 0.215 | 0.225 | 0.280 | 0.270 | 0.161 | **1.68** |
| *ribF* | 0.184 | 0.203 | 0.250 | 0.232 | 0.137 | **1.69** |
| *rpsE* | 0.096 | 0.121 | 0.146 | 0.121 | 0.071 | **1.70** |
| *murD* | 0.189 | 0.212 | 0.261 | 0.238 | 0.140 | **1.70** |
| *rpsC* | 0.113 | 0.113 | 0.144 | 0.143 | 0.083 | **1.73** |
| *lpxD* | 0.229 | 0.213 | 0.275 | 0.291 | 0.166 | **1.75** |
| *hisC* | 0.154 | 0.143 | 0.187 | 0.198 | 0.111 | **1.79** |
| *rpmA* | 0.120 | 0.118 | 0.152 | 0.154 | 0.086 | **1.79** |
| *qacE* | 0.123 | 0.128 | 0.163 | 0.158 | 0.088 | **1.80** |
| *gloB* | 0.285 | 0.295 | 0.377 | 0.367 | 0.203 | **1.81** |
| *rnpA* | 0.268 | 0.218 | 0.296 | 0.346 | 0.191 | **1.81** |
| *hisG* | 0.051 | 0.038 | 0.053 | 0.066 | 0.036 | **1.82** |
| *ureB* | 0.148 | 0.181 | 0.226 | 0.193 | 0.103 | **1.87** |
| *speB* | 0.149 | 0.144 | 0.191 | 0.195 | 0.103 | **1.90** |
| *dnaQ* | 0.217 | 0.224 | 0.292 | 0.285 | 0.150 | **1.91** |
| *acpP* | 0.072 | 0.064 | 0.087 | 0.095 | 0.049 | **1.93** |
| *rpsT* | 0.259 | 0.304 | 0.386 | 0.341 | 0.177 | **1.93** |
| *lptA* | 0.426 | 0.495 | 0.631 | 0.561 | 0.290 | **1.93** |
| *tpiA* | 0.294 | 0.257 | 0.351 | 0.388 | 0.201 | **1.93** |
| *apt* | 0.133 | 0.135 | 0.178 | 0.176 | 0.090 | **1.96** |
| *rpmE* | 0.128 | 0.183 | 0.225 | 0.170 | 0.085 | **1.99** |
| *glyQ* | 0.148 | 0.173 | 0.221 | 0.197 | 0.099 | **1.99** |
| *rplO* | 0.202 | 0.151 | 0.218 | 0.270 | 0.135 | **2.01** |
| *rppH* | 0.138 | 0.123 | 0.171 | 0.185 | 0.091 | **2.04** |
| *ygbF* | 0.264 | 0.301 | 0.393 | 0.356 | 0.172 | **2.07** |
| *tolR* | 0.288 | 0.377 | 0.481 | 0.391 | 0.184 | **2.13** |
| *yigL* | 0.230 | 0.203 | 0.287 | 0.314 | 0.145 | **2.17** |
| *rpsS* | 0.042 | 0.052 | 0.067 | 0.058 | 0.026 | **2.20** |
| *rplL* | 0.185 | 0.166 | 0.236 | 0.255 | 0.115 | **2.21** |
| *rpsP* | 0.214 | 0.177 | 0.259 | 0.296 | 0.133 | **2.22** |
| *rlpB* | 0.444 | 0.501 | 0.682 | 0.625 | 0.263 | **2.38** |
| *lptC* | 0.456 | 0.547 | 0.733 | 0.643 | 0.269 | **2.39** |
| *lpxA* | 0.081 | 0.061 | 0.096 | 0.116 | 0.046 | **2.55** |
| *bfr* | 0.252 | 0.198 | 0.312 | 0.367 | 0.137 | **2.67** |
| *atpH* | 0.310 | 0.344 | 0.489 | 0.454 | 0.165 | **2.74** |
| *hspQ* | 0.115 | 0.066 | 0.121 | 0.171 | 0.060 | **2.86** |
| *slyA* | 0.129 | 0.124 | 0.190 | 0.194 | 0.063 | **3.09** |
| *fabZ* | 0.053 | 0.020 | 0.054 | 0.087 | 0.019 | **4.58** |
