# Supplementary material for: Unprecedented loss of ammonia assimilation capability in a urease-encoding bacterial mutualist
Source: BMC Genomics. 2010 Dec 2;11:687. doi: 10.1186/1471-2164-11-687 (PMC3017870; doi:10.1186/1471-2164-11-687)
Supplement: Additional File 3 — Phylogenetic analysis of Blochmannia vafer. Description of phylogenetic analyses of the three sequenced Blochmannia. [file 1471-2164-11-687-S3.DOC]

**Additional File 3.**

**Phylogenetic analysis of *Blochmannia vafer*.**

Previous analyses of endosymbiont genes suggested that *B. vafer* and *B. floridanus* are more closely related to each other than either is to *B. pennsylvanicus* [1]. However, some genes showed conflicting signals or poor resolution. To resolve the relationships among these three endosymbiont taxa, we analyzed 12 highly conserved (>85% amino acid identity among all three species) protein-coding genes spanning several functional categories: three ATP synthase subunits (*atpB*, *atpD*, and *atpE*), cold shock protein *cspC*, serine protease *degQ*, chaperone *dnaK*, cell shape-determining protein *mreB*, ribose-phosphate pyrophosphokinase *prs*, transcription termination factor *rho*, and three ribosomal proteins (*rpsJ, rpsK,* and *rpsL*). Using *Sodalis glossinidius* (NC_007712) as an outgroup, we constructed single-gene trees using Bayesian phylogenetic methods. For each tree, we used the TransAlign PERL script [2] to align translated nucleotide sequences in ClustalX [3] and then back-translate the alignments for phylogenetic analysis using MrBayes v 3.1.2 [4]. We partitioned first plus second codon positions separately from third codon positions, and parameters for these two partitions were estimated independently. Based on a comparison of likelihood scores using the Akaike Information Criterion in jModelTest [5, 6], we used a general time reversible (GTR) substitution model, and rates were allowed to vary among sites according to a gamma distribution, with a proportion of invariant sites. Starting from a random tree, four Markov chain Monte Carlo (MCMC) chains were run. Trees were sampled every 100 generations for 1,000,000 generations, and the consensus tree was generated after a burn-in of 9,000 trees.

Ten of the twelve gene trees placed *B. vafer* and *B. floridanus* as each other’s closest relatives, with posterior probabilities ranging from 63-100%. By contrast, the *cspC* tree grouped *B. floridanus* and *B. pennsylvanicus* with a posterior probability of 52%, whereas the *atpE* tree grouped *B. vafer* and *B. pennsylvanicus* with a posterior probability of 98%. These two genes were the shortest of the ten, therefore they may not contain enough sequence information to accurately resolve the phylogenetic relationships of the three taxa. In addition, *atpE* was the only gene out of 570 to give a negative value in the evolutionary rates comparison (Additional File 2), meaning that this was the only gene to violate the underlying assumption that *B. pennsylvanicus* is the more distant sequence of the three. Based on the topology shared by the majority of the single gene trees, we infer that *B. vafer* is more closely related to *B. floridanus* than to *B. pennsylvanicus*.

1. Degnan PH, Lazarus AB, Brock CD, Wernegreen JJ: **Host-symbiont stability and fast evolutionary rates in an ant-bacterium association: cospeciation of *Camponotus* species and their endosymbionts, Candidatus *Blochmannia***. *Syst Biol* 2004, **53**(1):95-110.

2. Bininda-Emonds OR: **transAlign: using amino acids to facilitate the multiple alignment of protein-coding DNA sequences**. *BMC Bioinformatics* 2005, **6**:156.

3. Chenna R, Sugawara H, Koike T, Lopez R, Gibson TJ, Higgins DG, Thompson JD: **Multiple sequence alignment with the Clustal series of programs**. *Nucleic Acids Res* 2003, **31**(13):3497-3500.

4. Ronquist F, Huelsenbeck JP: **MrBayes 3: Bayesian phylogenetic inference under mixed models**. *Bioinformatics* 2003, **19**(12):1572-1574.

5. Guindon S, Gascuel O: **A simple, fast, and accurate algorithm to estimate large phylogenies by maximum likelihood**. *Syst Biol* 2003, **52**(5):696-704.

6. Posada D: **jModelTest: phylogenetic model averaging**. *Mol Biol Evol* 2008, **25**(7):1253-1256.
