# Supplementary material for: Unprecedented loss of ammonia assimilation capability in a urease-encoding bacterial mutualist
Source: BMC Genomics. 2010 Dec 2;11:687. doi: 10.1186/1471-2164-11-687 (PMC3017870; doi:10.1186/1471-2164-11-687)
Supplement: Additional File 4 — Gap closure. Detailed methods used to close two gaps in the B. vafer genome assembly. [file 1471-2164-11-687-S4.DOC]

**Additional File 4.**

**Gap closure**

To close the second gap, we examined the flanking sequence from the Velvet assembly and discovered 18 bp of identical sequence at the ends of both contigs. This sequence comprises part of an inverted repeat of the palindrome that impeded Sanger sequencing and Velvet assembly at this location. We manually joined the contigs together based on the 18 bp overlap and confirmed the resulting sequence by aligning the full Illumina read dataset using the Mosaik alignment program (<http://bioinformatics.bc.edu/marthlab/Mosaik>). The resulting coverage (averaging 470x) confirmed the manually closed gap sequence.

For the third gap, assembly of Sanger sequencing reads extended the end of one contig by 18 bp. We then aligned the full set of Illumina reads against this contig using Mosaik. In Consed, we identified reads that aligned to the contig but also extended past the end. We added the consensus sequence of these reads to the end of the contig and then used this extended contig as a reference sequence for the next Mosaik alignment. Five rounds of this alignment and extension process, each of which added 4-7 bp to the end of the contig, generated sequence that overlapped with the end of the other contig and spanned the gap. We checked the accuracy of the gap sequence by aligning the full Illumina read dataset. The resulting coverage (averaging 626x) confirmed the manually closed gap sequence.
